# Role of the AP-5 adaptor protein complex in late endosome-to-Golgi retrieval

**DOI:** 10.1371/journal.pbio.2004411

**Published:** 2018-01-30

**Authors:** Jennifer Hirst, Daniel N. Itzhak, Robin Antrobus, Georg H. H. Borner, Margaret S. Robinson

**Affiliations:** 1 University of Cambridge, Cambridge Institute for Medical Research, Cambridge, United Kingdom; 2 Max-Planck Institute of Biochemistry, Martinsried, Germany; UT Southwestern Medical Center, United States of America

## Abstract

The AP-5 adaptor protein complex is presumed to function in membrane traffic, but so far nothing is known about its pathway or its cargo. We have used CRISPR-Cas9 to knock out the AP-5 ζ subunit gene, AP5Z1, in HeLa cells, and then analysed the phenotype by subcellular fractionation profiling and quantitative mass spectrometry. The retromer complex had an altered steady-state distribution in the knockout cells, and several Golgi proteins, including GOLIM4 and GOLM1, were depleted from vesicle-enriched fractions. Immunolocalisation showed that loss of AP-5 led to impaired retrieval of the cation-independent mannose 6-phosphate receptor (CIMPR), GOLIM4, and GOLM1 from endosomes back to the Golgi region. Knocking down the retromer complex exacerbated this phenotype. Both the CIMPR and sortilin interacted with the AP-5–associated protein SPG15 in pull-down assays, and we propose that sortilin may act as a link between Golgi proteins and the AP-5/SPG11/SPG15 complex. Together, our findings suggest that AP-5 functions in a novel sorting step out of late endosomes, acting as a backup pathway for retromer. This provides a mechanistic explanation for why mutations in AP-5/SPG11/SPG15 cause cells to accumulate aberrant endolysosomes, and highlights the role of endosome/lysosome dysfunction in the pathology of hereditary spastic paraplegia and other neurodegenerative disorders.

## Author summary

Eukaryotic cells contain multiple membrane-bound compartments, each with a distinct function and molecular composition. Proteins are transported from one compartment to another by vesicular carriers. Formation of these carriers requires coat proteins, which both shape the membrane into a vesicle and select the proteins that are to be included as cargo. In many cases, cargo selection is facilitated by an adaptor protein (AP) complex, of which 5 have been identified. The most recently identified complex, AP-5, localises to a late endosomal/lysosomal compartment, and patients with mutations in AP-5 have a form of hereditary spastic paraplegia characterised by aberrant lysosomes. However, the precise function of AP-5, including its cargo and its pathway, has until now been unclear. In the present study, we have used unbiased subcellular proteomics to look for changes in the localisation of thousands of different proteins in cells from which AP-5 has been deleted by gene editing. We found that there are defects in the retrieval of several proteins from late endosomes back to the Golgi apparatus. Thus, we propose that AP-5 facilitates a novel late-acting retrieval pathway, which contributes to normal lysosomal homeostasis.

## Introduction

Adaptor protein (AP) complexes are a family of 5 evolutionarily ancient heterotetramers [1], which facilitate the transport of cargo from one membrane compartment of the cell to another. The most recently discovered AP complex, AP-5 [2], had escaped detection for over 10 years, because its subunits were too divergent to be identified using sequence-based tools like BLAST. However, AP-5 is predicted to be structurally very similar to APs 1–4, even though in its native form it exists as a heterohexamer rather than a heterotetramer. Its two additional subunits are encoded by the SPG11 and SPG15 genes, and they are essential for the stability and membrane association of the whole complex [2,3].

Understanding the function of AP-5 has been challenging. It is expressed at relatively low levels (only about 10,000 copies in a HeLa cell, compared with about 300,000–1,000,000 copies for APs 1, 2, or 3) [4,5], and it has been lost from several model organisms, including *Drosophila melanogaster*, *Caenorhabditis elegans*, and *Saccharomyces cerevisiae* [6]. However, it is clearly important in humans, because mutations in its ζ subunit, encoded by the AP5Z1 gene (aka SPG48), cause hereditary spastic paraplegia (HSP) [7], as do mutations in either SPG11 or SPG15 (SPG is an acronym for spastic paraplegia gene). In all three cases, the disorder is classified as a complicated form of HSP, with various neurological abnormalities in addition to the typical degeneration of long corticospinal axons, which is a defining feature of all forms of HSP [8].

The AP-5/SPG11/SPG15 complex localises to late endosomes and lysosomes [2,3], and fibroblasts from HSP patients with mutations in any of the 3 genes contain aberrant endolysosomes filled with undigested material [9–11]. Some of the other forms of HSP, including those caused by mutations in the SPG4, SPG8, or SPG31 genes, are also characterised by lysosomal abnormalities [12], suggesting that lysosome dysfunction may be a common feature of the disorder and may play a causative role. However, in the case of the AP-5/SPG11/SPG15 complex, the molecular mechanisms underlying this phenotype are unclear, because the function of AP-5 and its associated proteins is still unknown.

The homology between AP-5 and the other APs suggests that it may be involved in cargo recognition, but this has not been demonstrated, and the highly conserved cargo binding sites found in the other AP complexes are absent in AP-5. One possibility would be to look for AP-5–dependent cargo by carrying out conventional binding assays. However, previous studies have shown that adaptor-cargo interactions are generally low affinity, transient, and dependent upon the presence of other factors and/or the conformation state of the complex [13]. Therefore, we used an alternative approach: subcellular fractionation profiling combined with quantitative mass spectrometry to identify proteins whose subcellular distribution is affected by the presence or absence of AP-5.

## Results

### Whole cell proteomics

**Fig 1:**
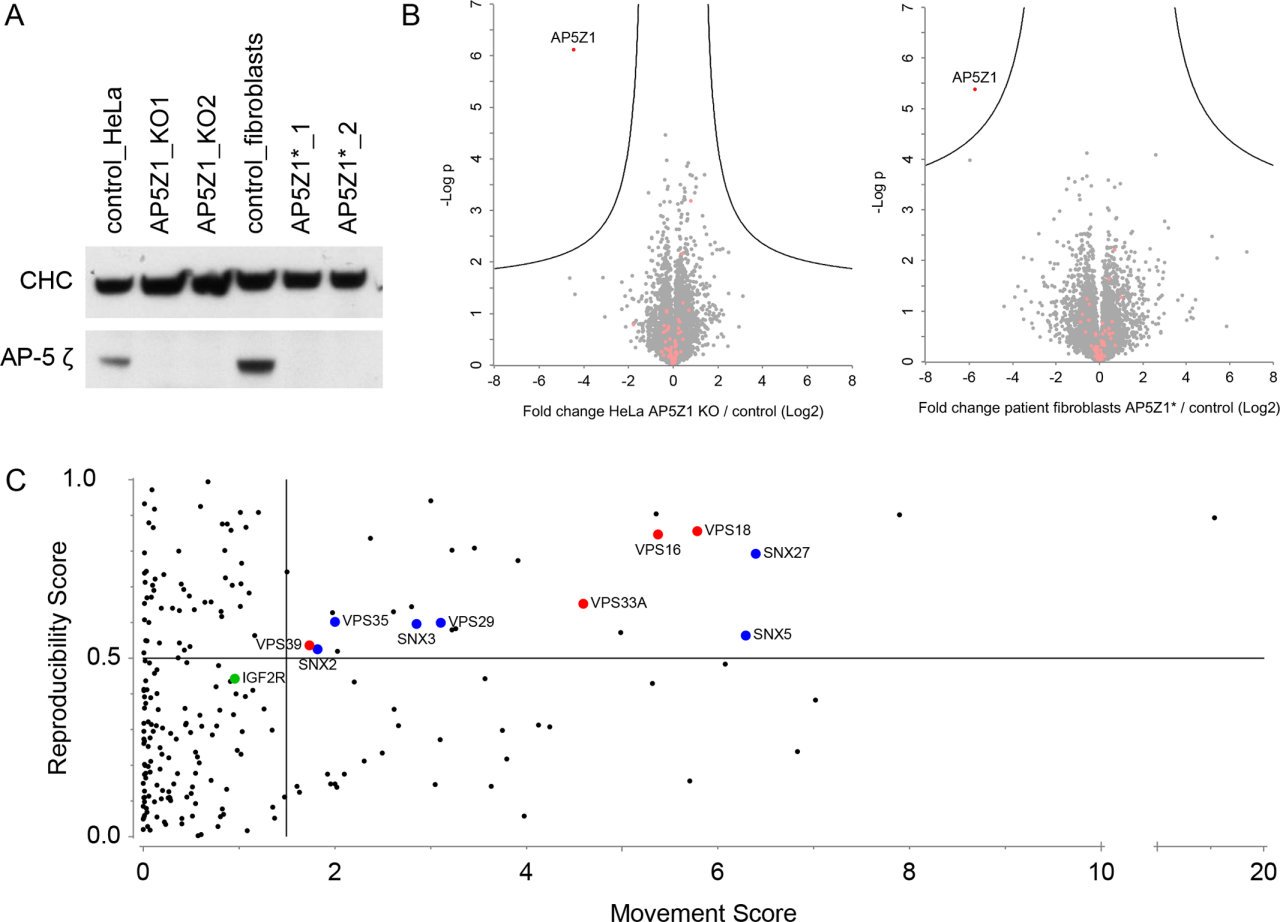
Comparison of control and AP5Z1-deficient cells. (A) Whole cell lysates from control HeLa cells and HeLa AP5Z1 knockout lines (KO1 and KO2) (first three lanes), and control and AP5Z1 patient-derived fibroblast lines AP5Z1*_1 p.(Q587*) and AP5Z1* p.(R138*);(p.W441*) [9,15] (second three lanes). In the knockout and patient lines, there is complete loss of AP-5 ζ. CHC was included as a loading control. (B) Global proteome analysis. Left panel, HeLa cells: AP5Z1 knockout versus controls. Right panel, human fibroblasts: AP5Z1* versus matched controls. Whole cell lysates were analysed by label-free quantification mass spectrometry. In both cell types, over 7,500 proteins were quantified. The x-axis shows the log_2_-fold change between AP-5–deficient and control cells; the y-axis shows the −log_10_ *p*-value of significance (2-sided *t* test). The ‘volcano’ lines indicate the significance threshold (FDR 0.12). For HeLa cells, 3 replicates from control cells and 3 replicates each from 2 independent AP5Z1-knockout cell lines (6 total) were compared (*n* = 3/6). For fibroblasts, 2 cell lines from patients with null mutations in AP5Z1 were compared to 2 matched control cell lines, each in duplicate (i.e., *n* = 4). Lysosomal proteins are indicated (salmon-coloured dots); their abundance does not change significantly in AP-5–deficient cells. Data can be found in S2 Data (HeLa intensity data and Fibroblast intensity data); the plots were generated using Perseus software. (C) Analysis of protein movement on dynamic organellar maps. Control HeLa cells and the 2 independent AP5Z1 knockout lines were subjected to proteomics-based organellar mapping, each in triplicate (see also S1 Fig). Maps from control and AP5Z1 knockout cells were then compared to detect proteins undergoing shifts in subcellular localization, in which the reproducibility score is a measure of the correlation between replicates of the same clone and between the 2 different AP5Z1 knockout clones. For each protein, the M and R of movement were calculated. Significantly shifting proteins have high M and R scores and are located in the top right quadrant of the MR plot; they are highly enriched in endosomal proteins. The estimated FDR was <0.23 at the cutoffs indicated by the vertical (M score threshold) and horizontal (R score threshold) lines. Subunits of the HOPS (red) and retromer (blue) complexes are highlighted. The cation-independent mannose 6-phosphate receptor (IGFR2R) is a marginal hit (significant at FDR 0.25). The figure focuses on the area of the MR plot in which significant changes are located (R score > 0); see S1 Data (MR plot data) for a complete list of all 2,046 profiled proteins. AP, adaptor protein; CHC, clathrin heavy chain; KO, knockout; M, magnitude; R, reproducibility.

To investigate the phenotype of AP-5 deficiency, we first looked for changes in global protein expression levels using label-free mass spectrometric quantification [14]. Two independent knockouts of the AP-5 ζ subunit gene, AP5Z1, were made in HeLa cells using CRISPR-Cas9 (AP5Z1_KO1 and AP5Z1_KO2; Fig 1A) and compared with wild-type cells. In addition, fibrobasts from 2 unrelated patients with loss-of-function mutations in AP5Z1 were compared with matched controls [9] (Fig 1A). With the exception of AP5Z1 itself, there was little difference in the relative amounts of the >7,500 proteins identified in the HeLa cell lines or in the >7,500 proteins identified in the patient fibroblast lines (Fig 1B). Because AP-5 localises to a late endocytic compartment [2,3], and loss of AP-5 causes cells to accumulate aberrant endolysosomes [9], we anticipated that there might be problems with lysosomal degradation and/or effects on proteins associated with late endosomes and lysosomes. However, levels of lysosomal proteins (coloured salmon in Fig 1B) were essentially unaffected in both HeLa cells and fibroblasts. Because fibroblasts are not very tractable for biochemical analyses, owing to their genetic heterogeneity, slow growth, and resistance to transfection, we focused on the HeLa knockout cell lines for our further investigations.

### Dynamic organellar mapping

Although there were no obvious changes in protein abundance when the knockout and mutant cells were compared with controls, AP-5 is likely to have a role in protein sorting; thus, we next investigated whether AP-5 ablation causes changes in the subcellular localisation of proteins. For this, we applied a proteomic technique developed in the Borner lab called “Dynamic Organellar Maps” [5,16]. This approach combines subcellular fractionation with quantitative mass spectrometry profiling to determine the compartment associations of proteins. Comparisons of organellar maps made under different conditions reveal shifts in the fractionation profile of proteins in an unbiased manner.

Control HeLa cells and both of the AP5Z1 knockout lines were analysed in triplicate, and 2,046 proteins were profiled across all 9 maps. Principal component analysis showed highly resolved organellar clusters, with similar maps for controls and knockouts (S1 Fig). To detect proteins whose subcellular localisation (i.e., fractionation profile) was changed by AP-5 ablation, we performed a sensitive shift analysis, scoring each protein for the magnitude and reproducibility of its movement across maps (see Materials and methods for details). This identified 26 candidate shifting proteins (Fig 1C; S1 Data, “MR plot data”). Strikingly, 15 of them were known or predicted endosomal proteins (GO-term “endosome membrane” >10-fold enriched, *p* < 1.5*10^9^; S1 Data, “Enrichment of MR plot hits”). They included multiple subunits of the retromer complex and associated sorting nexins, which are involved in endosome–to–trans-Golgi network (TGN) recycling (VPS29, VPS35, SNX2, SNX3, SNX5, SNX27), as well as multiple subunits of the HOPS complex, which is involved in endosome-lysosome fusion (VPS16, VPS18, VPS33A, VPS39). The cation-independent mannose 6-phosphate receptor (CIMPR; gene name IGF2R) was also identified as a marginal hit.

Inspection of individual map positions revealed that these proteins were still predicted to be mostly endosomal in the AP-5 knockout cells, but they fractionated more closely with lysosomes than in control cells (S1 Fig). Although the biological interpretation of the shifts is not straightforward, the movements of the proteins on the maps were indicative of an association with a larger or denser compartment in the AP-5 knockouts. The organellar mapping data are thus consistent with the previously observed AP-5 endolysosomal swelling phenotype [9] and identify HOPS and retromer plus sorting nexins as major candidate protein sorting complexes responding to AP-5 ablation. Because we had previously observed changes in retromer localisation following small interfering RNA (siRNA)-mediated depletion of AP-5 [2,3], we decided to investigate the relationship between AP-5 and retromer further.

### AP-5 and retromer

**Fig 2:**
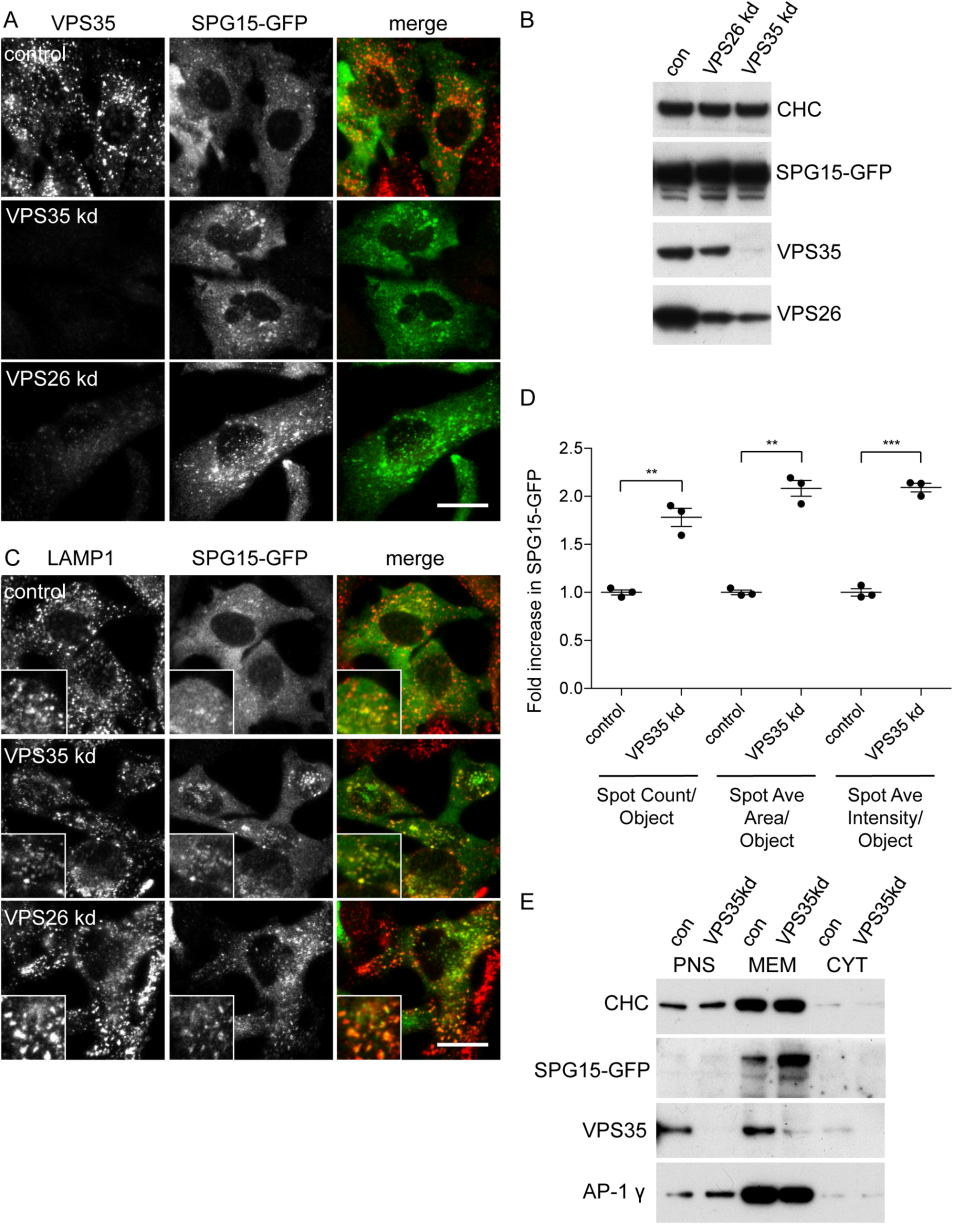
Effect of retromer knockdown on SPG15-GFP localisation. (A) Indirect immunofluorescence microscopy of SPG15-GFP–expressing cells depleted of either VPS35 or VPS26 by siRNA, and double labelled with antibodies against VPS35 and GFP. The GFP puncta are larger and brighter when VPS35 or VPS26 is depleted. Scale bar: 20 μm. (B) Whole cell homogenates of control, VPS35 knockdown, and VPS26 knockdown cells. VPS35 was knocked down with >95% efficiency and VPS26 with >80% efficiency. The knockdown of VPS26 reduces the protein levels of VPS35 and vice versa. SPG15-GFP is unchanged. (C) Indirect immunofluorescence microscopy of SPG15-GFP–expressing control, VPS35 knockdown, and VPS26 knockdown cells double labelled with antibodies against LAMP1 and GFP. There is substantial colocalisation of the 2 proteins. Scale bar: 20 μm. (D) Quantification of the increase in SPG15-GFP fluorescence using an automated microscope and a SpotDetector Bioapplication. The VPS35 knockdown causes increases in spot count per object (1.78 ± 0.09), spot average area per object (2.08 ± 0.08), and spot average intensity per object (2.09 ± 0.04). More than 1,500 cells were scored per knockdown condition (3 independent repeats); error bars indicate SEM. Because the number of spots per object goes up, the increases in fluorescence brightness are unlikely to be due to spot clustering. The raw data can be found in S4 Data. (E) Membrane-containing pellet and soluble cytosol fractions from SPG15-GFP–expressing cells, either control or after VPS35 knockdown. Equal amounts of protein were loaded into the PNS, MEM, and CYT lanes. Thus, in terms of starting volume, the MEM fraction was overloaded by about 5-fold. Knocking down retromer resulted in a 1.79 ± 0.23-fold increase in membrane-associated SPG15-GFP (3 biological repeats). CHC, clathrin heavy chain; con, control; CYT, cytosol; MEM, membrane; PNS, postnuclear supernatant; siRNA, small interfering RNA; SPG, spastic paraplegia gene.

We began by using immunofluorescence microscopy to investigate the effect of retromer knockdown on AP-5 and its partners, SPG11 and SPG15. Because of signal-to-background problems with antibodies against endogenous subunits, which are expressed at very low levels [6], we carried out these experiments on cells expressing GFP-tagged SPG15 under the control of its endogenous promoter [7]. We found that knocking down either VPS26 or VPS35 caused the tagged SPG15 to appear much brighter (Fig 2A), even though western blotting showed that there was no increase in the total amount of the construct (Fig 2B), and the construct still localised to a LAMP1-positive compartment (Fig 2C). Quantification using automated microscopy, which objectively samples thousands of cells, revealed an approximately 2-fold increase in the size and intensity of SPG15-GFP “spots” (Fig 2D). Although this increase could represent coalescence of smaller compartments, the number of quantified spots per object (i.e., per cell) did not decrease but in fact increased, indicating that more SPG15-GFP was being recruited onto membranes. Similarly, western blotting of cell homogenates, which had been separated into membranes and cytosol by ultracentrifugation, showed that knocking down VPS35 caused an increase in membrane-associated SPG15-GFP (Fig 2E). These findings suggest that, in the absence of retromer, the AP-5/SPG11/SPG15 complex is somehow harnessed more efficiently in order to compensate and thus that retromer and AP-5/SPG11/SPG15 might be functioning in the same or related pathways.

**Fig 3:**
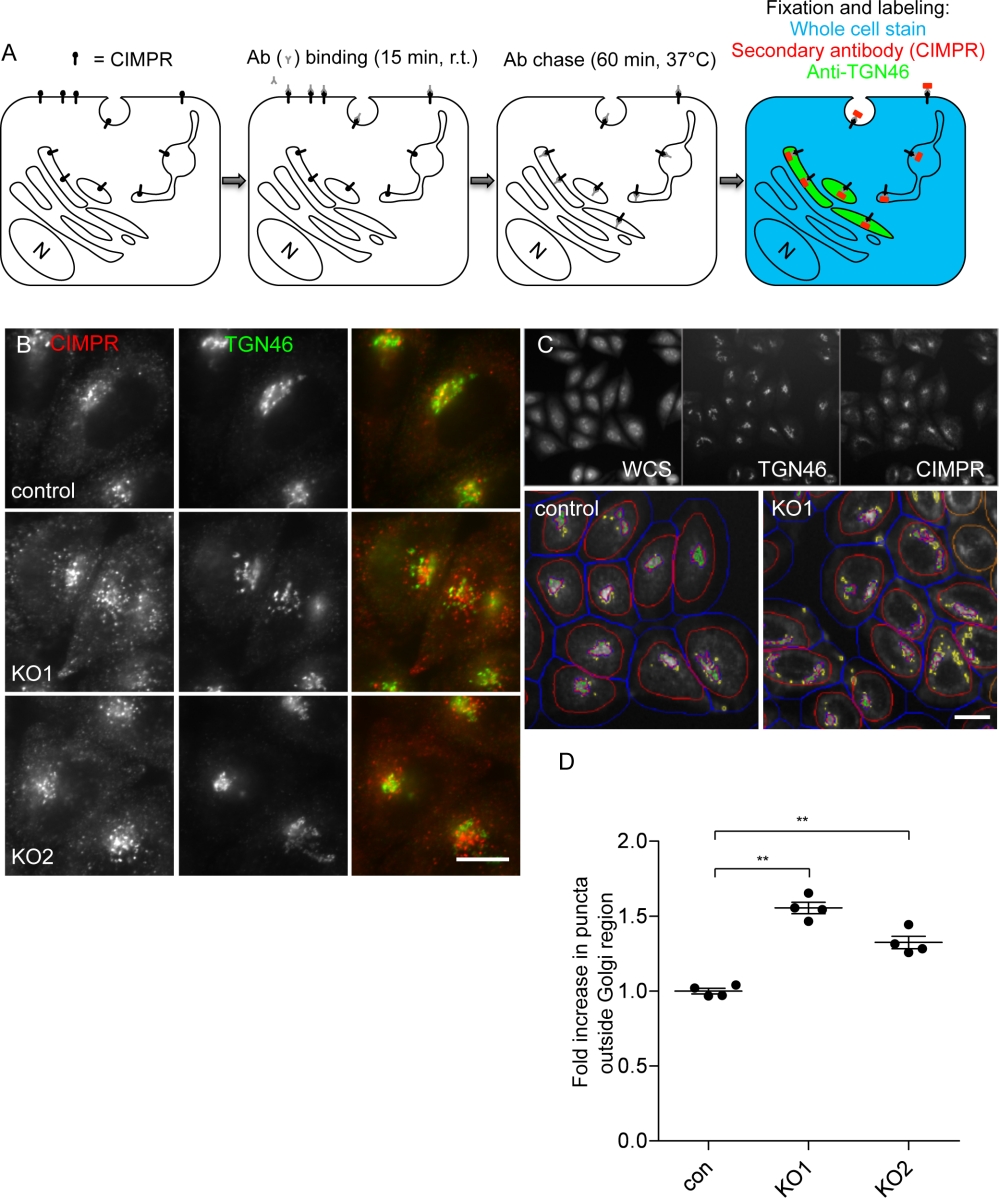
CIMPR trafficking in control and AP5Z1 knockout cells. (A) Schematic representation of the CIMPR retrieval assay, which follows the trafficking of endogenous CIMPR. (B) Indirect immunofluorescence microscopy of control and AP5Z1 knockout cells, which were pulse chased with an antibody against CIMPR, as shown in Fig 3A. In both knockout lines, there was reduced retrieval of anti-CIMPR back to the juxtanuclear region, as defined by TGN46 labelling. These results are consistent with the identification of AP-5 ζ (KIAA0415) as a potential hit in a genome-wide screen for proteins involved in endosomal retrieval [17]. Scale bar: 20 μm. (C) Quantification of the retrieval defect using an Arrayscan automated microscope. The whole cell stain allowed a mask to be drawn around the cells (red), an offset line was added to ensure that the whole cell was captured (blue), and the anti-TGN46 allowed a mask to be drawn around the TGN (pink). The CIMPR that failed to be retrieved back to the TGN is shown in yellow. Scale bar: 20 μm. (D) Analysis of the data from the Arrayscan microscope, using a Colocalisation Bioapplication. The fold increase in CIMPR (Object Total Area) that failed to be retrieved back to the juxtanuclear was 1.55 ± 0.04 for AP5Z1_KO1 and 1.32 ± 0.04 for AP5Z1_KO2. More than 1,500 cells were scored per knockdown condition (4 independent repeats; error bars indicate SEM). The raw data can be found in S4 Data. Ab, antibody; AP, adaptor protein; CIMPR, cation-independent mannose 6-phosphate receptor; con, control; KO, knockout; TGN, trans-Golgi network; WCS, whole cell stain.

To test this possibility, we adapted a retrieval assay for the CIMPR, which was originally designed to study retromer function [17]. Cells were incubated at room temperature with an antibody against endogenous CIMPR, then warmed to 37°C for an hour to allow the antibody to be internalised. The cells were then fixed and triple labelled for the endocytosed antibody, the TGN region (using an antibody against TGN46), and the cell boundary (using a whole-cell stain) (Fig 3A). Both of the AP-5 knockout cell lines were significantly impaired in their ability to retrieve anti-CIMPR to the juxtanuclear region and showed less overlap with TGN46, although at this resolution it is not clear whether the antibody is in the TGN46 compartment or in another compartment in the same general vicinity (Fig 3B). The extent of overlap was quantified using an automated microscope (Fig 3C and Fig 3D).

**Fig 4:**
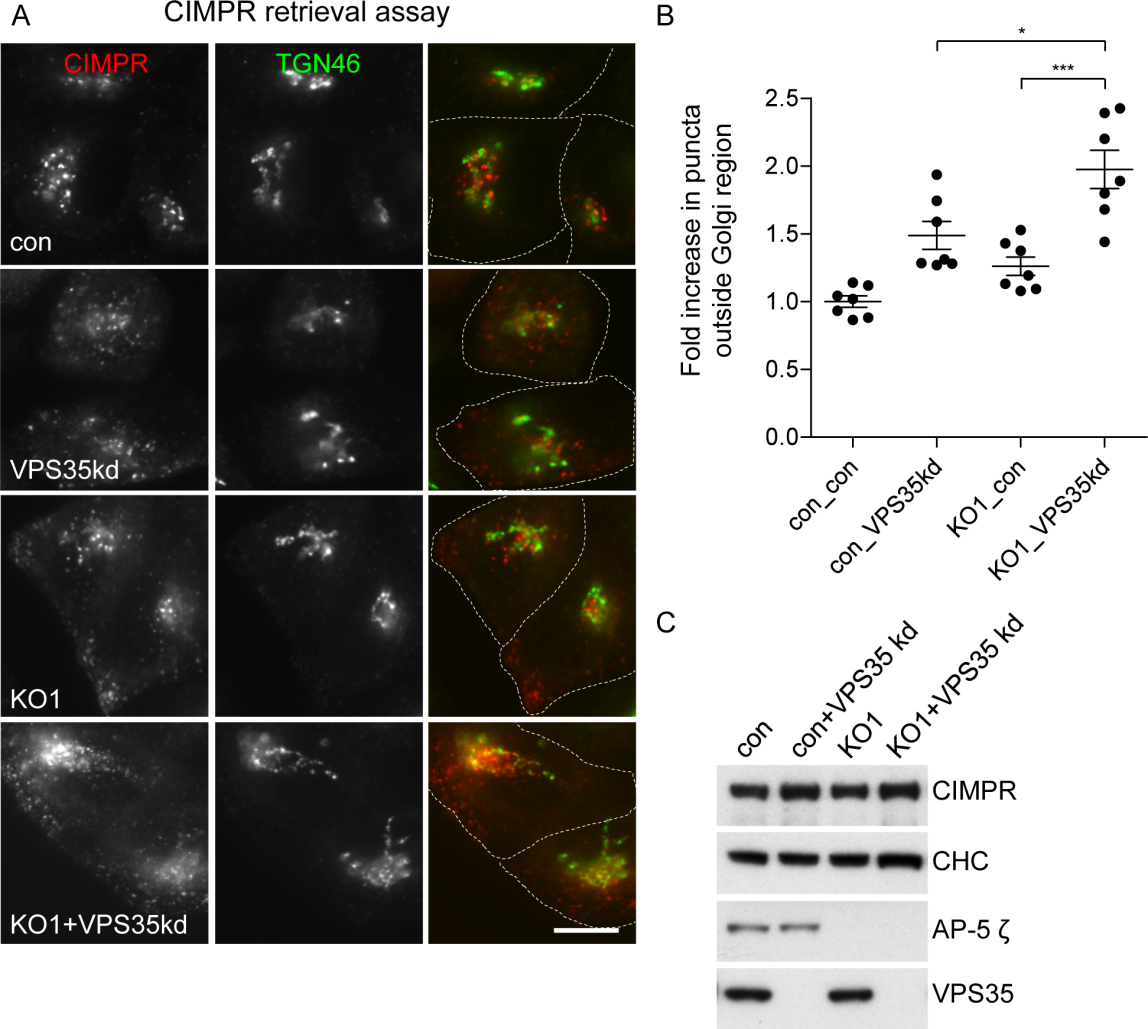
Effect of combined loss of retromer and AP-5 on CIMPR retrieval. (A) Immunofluorescence microscopy of control or AP5Z1 knockout cells depleted of VPS35 and pulse chased with anti-CIMPR, as shown in Fig 3A. Individually, the knockout of AP5Z1 or the knockdown of retromer caused a reduction in the retrieval of CIMPR back to the TGN region; this was further exacerbated when the knockout and knockdown were combined. The dotted lines indicate the boundaries of each cell. Scale bar: 20 μm. (B) Quantification of the retrieval defect of CIMPR, using the CX7 automated microscope and a Colocalisation Bioapplication. The increase in CIMPR (Total Object Count) that failed to be retrieved back to the TGN region was 1.48 ± 0.10 for VPS35 kd, 1.26 ± 0.07 for KO1, and 1.97 ± 0.14 for the combined KO1 plus VPS35 kd. More than 1,500 cells were scored per knockdown condition (7 independent repeats; error bars indicate SEM). The raw data can be found in S4 Data. (C) Whole cell lysates from control and AP5Z1 knockout lines. The steady-state levels of CIMPR are not significantly affected by loss of AP-5 or depletion of VPS35. In addition, the surface expression of CIMPR did not change substantially in the knockdown and knockout cells when compared to control, as determined by flow cytometry (VPS35kd 1.22 ± 0.36, KO1 1.12 ± 0.18, KO1+VPS35kd 1.25 ± 0.44). AP, adaptor protein; CHC, clathrin heavy chain; CIMPR, cation-independent mannose 6-phosphate receptor; con, control; KO, knockout; TGN, trans-Golgi network.

There was also impaired antibody retrieval to the juxtanuclear region when the VPS35 subunit of retromer was knocked down using siRNA (Fig 4A), in keeping with published results [17]. Importantly, this phenotype was even more pronounced when retromer was knocked down in AP5Z1 knockout cells (Fig 4A and Fig 4B). These differences were not due to effects on CIMPR expression levels nor to the amount of CIMPR on the cell surface, as determined by flow cytometry (Fig 4C). Together, our findings suggest that AP-5 and retromer may be working on parallel pathways to facilitate the retrieval of CIMPR from endosomes back to the TGN.

### Effect of AP-5 knockout on transport intermediates

**Fig 5:**
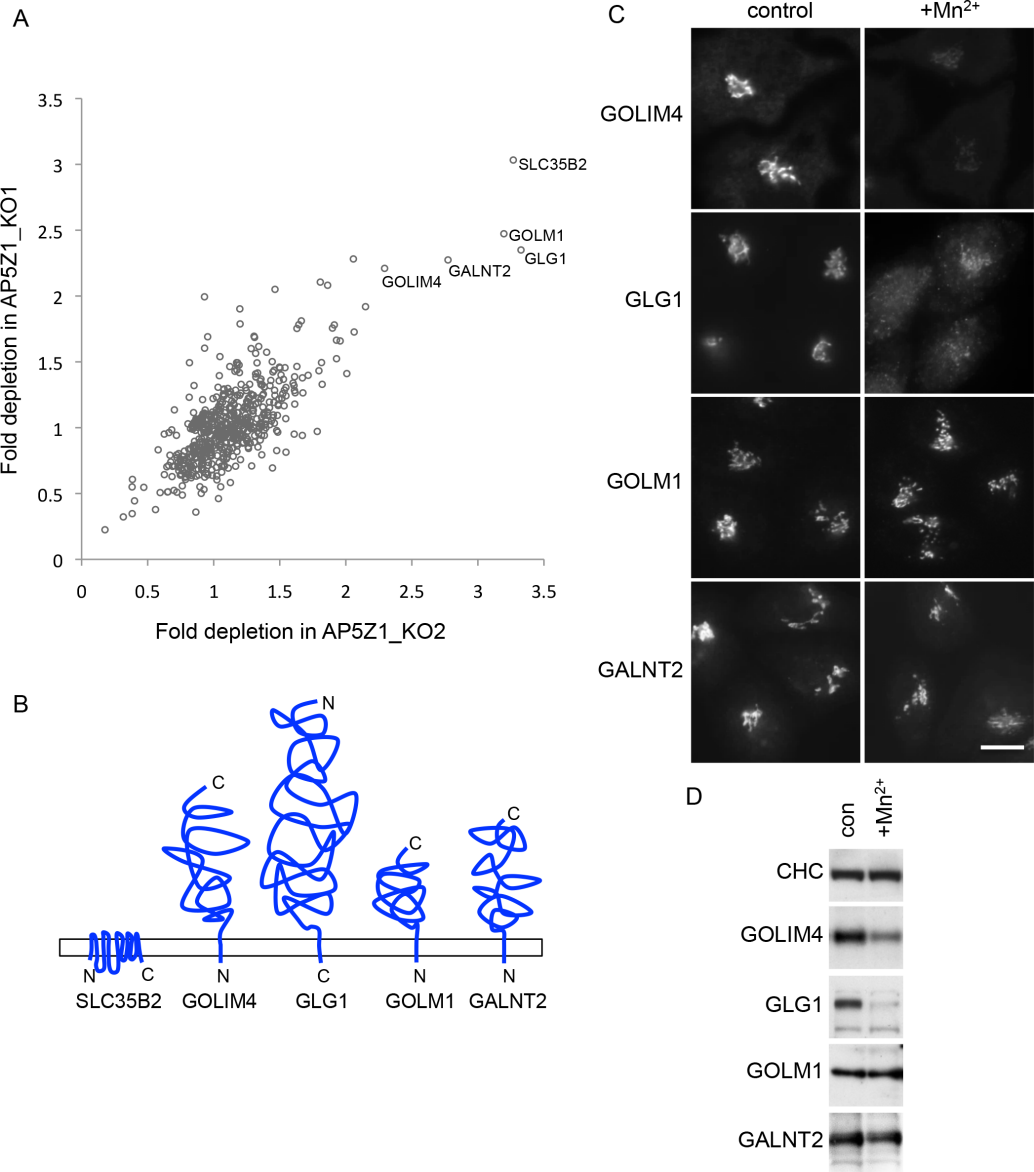
AP-5 knockout affects Golgi-localised proteins. (A) Scatterplot comparing proteins that were depleted in vesicle-enriched fractions from SILAC-labelled AP5Z1_KO1 and AP5Z1_KO2 cells. Although AP-5 was not detected in the vesicle-enriched fraction, we looked for proteins whose trafficking through vesicular carriers might be altered by the loss of AP-5. Results are based on 2 biological repeats and show 5 Golgi-associated transmembrane proteins that were consistently depleted in both independent AP5Z1 knockouts. Data can be found in S2 Data. (B) Schematic diagram showing the topologies of the Golgi-associated proteins that were identified as possible hits in Fig 5A. (C) Indirect immunofluorescence microscopy of GOLIM4, GLG1, GALNT2, and GOLM1. The addition of manganese (Mn^2+^) for 4 h results in the redistribution of both GOLIM4 and GLG1 away from the Golgi region of the cell. Scale bar: 20 μm. (D) Whole cell lysates from control and Mn^2+^-treated cells. The addition of Mn^2+^ reduces the GOLIM4 signal by 52% and the GLG1 signal by 89%, presumably due to lysosomal degradation and consistent with the diminished immunofluorescence signal. AP, adaptor protein; CHC, clathrin heavy chain; con, control; KO, knockout; Mn^2+^, manganese; SILAC, stable isotope labelling of amino acids in cell culture.

To search for other proteins whose trafficking might be affected by the loss of AP-5, we combined stable isotope labelling of amino acids in cell culture (SILAC) labelling with subcellular fractionation to isolate vesicle-enriched fractions from control and AP-5 knockout cells, which were then compared by quantitative mass spectrometry. These vesicle-enriched fractions are isolated from cell homogenates by differential centrifugation and contain a mixture of small membranous structures, including vesicles of many different types as well as other particles, such as ribosomes and proteasomes. We identified about 700 proteins across all 4 samples (two knockout lines, repeated twice; Fig 5A, S2 Data) and compiled a list of proteins that were consistently depleted more than 2-fold in the 2 knockout lines in both repeats. The top hits included 5 transmembrane proteins of varying topologies, all of which are reported to localise to the Golgi apparatus: SLC35B2, GOLIM4, GLG1, GOLM1, and GALNT2 [18–22] (Fig 5A and Fig 5B).

The identification of Golgi-localised proteins in this assay was surprising, because AP-5 localises to late endosomes and/or lysosomes, so we did not expect to find proteins residing in a relatively early secretory compartment. However, even though the 5 proteins are mainly resident in the Golgi at steady state, there is evidence that they can all traffic out of the Golgi to a later compartment and then recycle back again. GOLIM4 (aka GPP130, GIMPC, and GOLPH4) is probably the best characterised protein on our list. It was first described 20 years ago, when it was found to localise to the early Golgi but to have late Golgi posttranslational modifications, indicating that it cycles back and forth between early and late compartments [20]. GOLIM4 was also shown to redistribute to endosomes when cells were treated with pH-disrupting reagents, like chloroquine or monensin, but then to return to the Golgi when the drugs were removed [23,24]. Further studies showed that GOLIM4 acts as a receptor for Shiga toxin, facilitating its trafficking from endosomes back to the Golgi apparatus [25], but that this pathway can be blocked by the addition of manganese (Mn^2+^), which causes GOLIM4 to accumulate in late endosomes and lysosomes [26]. To determine whether any of our other hits might be Mn^2+^ sensitive, we treated cells with 500 μM MnCl_2_ and found that GLG1, but not GOLM1, GALNT2, or SLC35B2, showed a similar behaviour to GOLIM4 (Fig 5C and Fig 5D).

Another way of determining whether Golgi membrane proteins move to a later compartment and then return is to find out whether they are packaged as cargo into intracellular clathrin-coated vesicles (CCVs), which traffic back and forth between the TGN and endosomes. The major components of the coats on intracellular CCVs are clathrin and the AP-1 adaptor complex, and by knocking AP-1 sideways (i.e., rapidly redistributing it to mitochondria with a small molecule) [27] and then using mass spectrometry to look for differences in a CCV-enriched fraction, we can identify intracellular CCV cargo [28]. SLC35B2, GLG1, GOLM1, and GALNT2 were all depleted from the AP-1 knocksideways CCV fraction (2.3-, 1.9-, 2.2-, and 1.7-fold, respectively) [28]. Because AP-1 facilitates cycling between the TGN and endosomes, this indicates that these 4 proteins frequently move to endosomes, even though at steady state they are mainly in the Golgi. In contrast, GOLIM4 was only weakly affected by the AP-1 knocksideways [28]. However, its accumulation in endosomes in Mn^2+^-treated cells was reported to be clathrin dependent [29], indicating that it, too, can enter intracellular CCVs but that normally, there is relatively little of it in CCVs at steady state.

**Fig 6:**
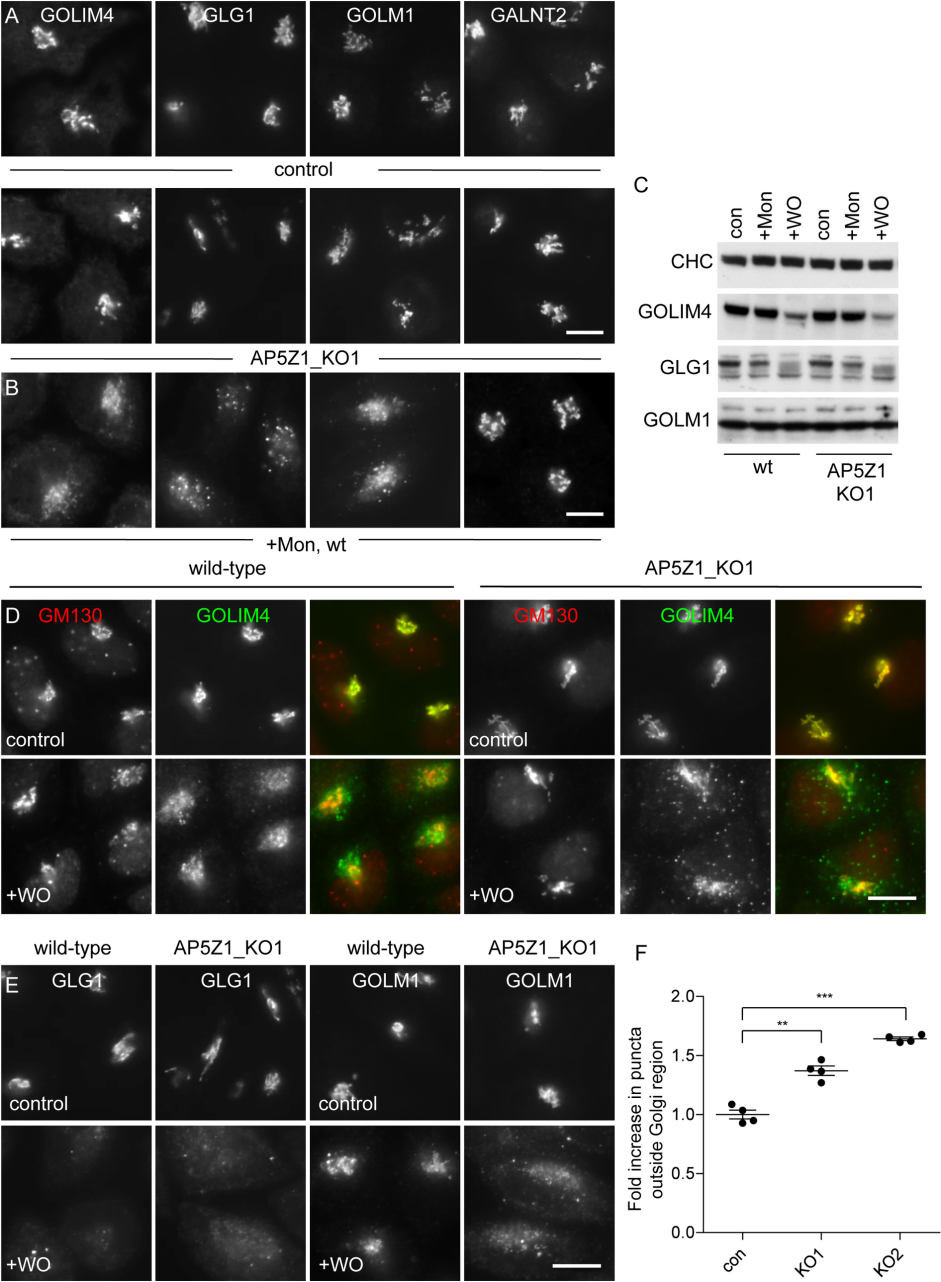
Effect of AP-5 knockout and monensin washout on the Golgi-localised proteins. (A) Indirect immunofluorescence microscopy of GOLIM4, GLG1, GOLM1, and GALNT2 in AP5Z1_KO cells. The localisation of the 4 proteins is unaffected by the knockout of AP5Z1 (compare with Fig 5C). Scale bar: 20 μm. (B) Indirect immunofluorescence microscopy of GOLIM4, GLG1, GOLM1, and GALNT2 following treatment with monensin for 90 min. GOLIM4, GLG1, and GOLM1 have redistributed away from the Golgi region. Scale bar: 20 μm. (C) Whole cell lysates of control and AP5Z1 knockout cells following treatment with monensin for 90 min and then a monensin washout for 2.25 h. GOLIM4 shows evidence of degradation after the washout in both control (54.5 ± 4.7%) and AP5Z1 knockout cells (44.0 ± 5.9%). Similar results were obtained for GLG1, but they were not quantifiable because of background bands. (D) Immunofluorescence microscopy of control and AP5Z1 knockout HeLa cells treated with monensin for 90 min followed by washout for 2.25 h. In the knockout, there is reduced retrieval of GOLIM4 back to the juxtanuclear region, where GM130 is located. The nuclear puncta labelled by the GM130 antibody are nonspecific. Scale bar: 20 μm. (E) Immunofluorescence microscopy of control and AP5Z1 knockout HeLa cells treated with monensin for 90 min followed by a washout for 2.25 h. Like GOLIM4, GOLM1 is impaired in its ability to be retrieved back towards the Golgi. GLG1 is not retrieved in either condition, and the diminished brightness correlates with its loss in western blots (Fig 6C). Scale bar: 20 μm. (F) Quantification of the retrieval defect was performed using an Arrayscan automated microscope and a Colocalisation Bioapplication using an adapted protocol that was designed for quantifying CIMPR retrieval (Fig 3C). The amount of GOLIM4 (Total Object Area) that was not retrieved towards the Golgi was 1.37 ± 0.04 for AP5Z1_KO1 and 1.64 ± 0.02 for AP5Z1_KO2, relative to control. More than 1,500 cells were scored per knockdown condition (3 independent repeats); error bars indicate SEM. The raw data can be found in S4 Data. AP, adaptor protein; CIMPR, cation-independent mannose 6-phosphate receptor; con, control; KO, knockout; Mon, monensin; WO, washout; wt, wild-type.

Probably the simplest and most versatile way of looking for Golgi escape and retrieval is to treat cells with a pH-disrupting drug like monensin or chloroquine. For reasons that are still unclear, raising the pH of acidic organelles causes many cycling proteins to become trapped in endosomes, although most then return to the Golgi when the drug is removed [30]. Thus, we investigated the localisation of the proteins upon monensin treatment and washout in both wild-type and AP-5 knockout cells. We found that in the absence of monensin, the proteins all localised normally to the Golgi in AP-5 knockout cells (Fig 6A). This is consistent with our organellar maps, in which the steady-state localisation of most proteins was unchanged by the AP-5 knockout (Fig 1 and S1 Fig). Treating the cells with monensin for 90 min caused GOLIM4, GLG1, and GOLM1 to adopt a more punctate and peripheral pattern (Fig 6B), presumably reflecting a move to endosomes [24]. This occurred without any loss in protein expression levels (Fig 6C). When the drug was then washed out for 2.25 h, GOLIM4 and GOLM1 returned to a juxtanuclear pattern, while GLG1 labelling was difficult to discern because of protein loss, presumably reflecting degradation in lysosomes (Fig 6C). Importantly, both GOLIM4 and GOLM1 were impaired in their ability to recycle back to the juxtanuclear region in the AP-5 knockout cells (Fig 6D and Fig 6E), and we were able to quantify this effect by automated microscopy (Fig 6F).

**Fig 7:**
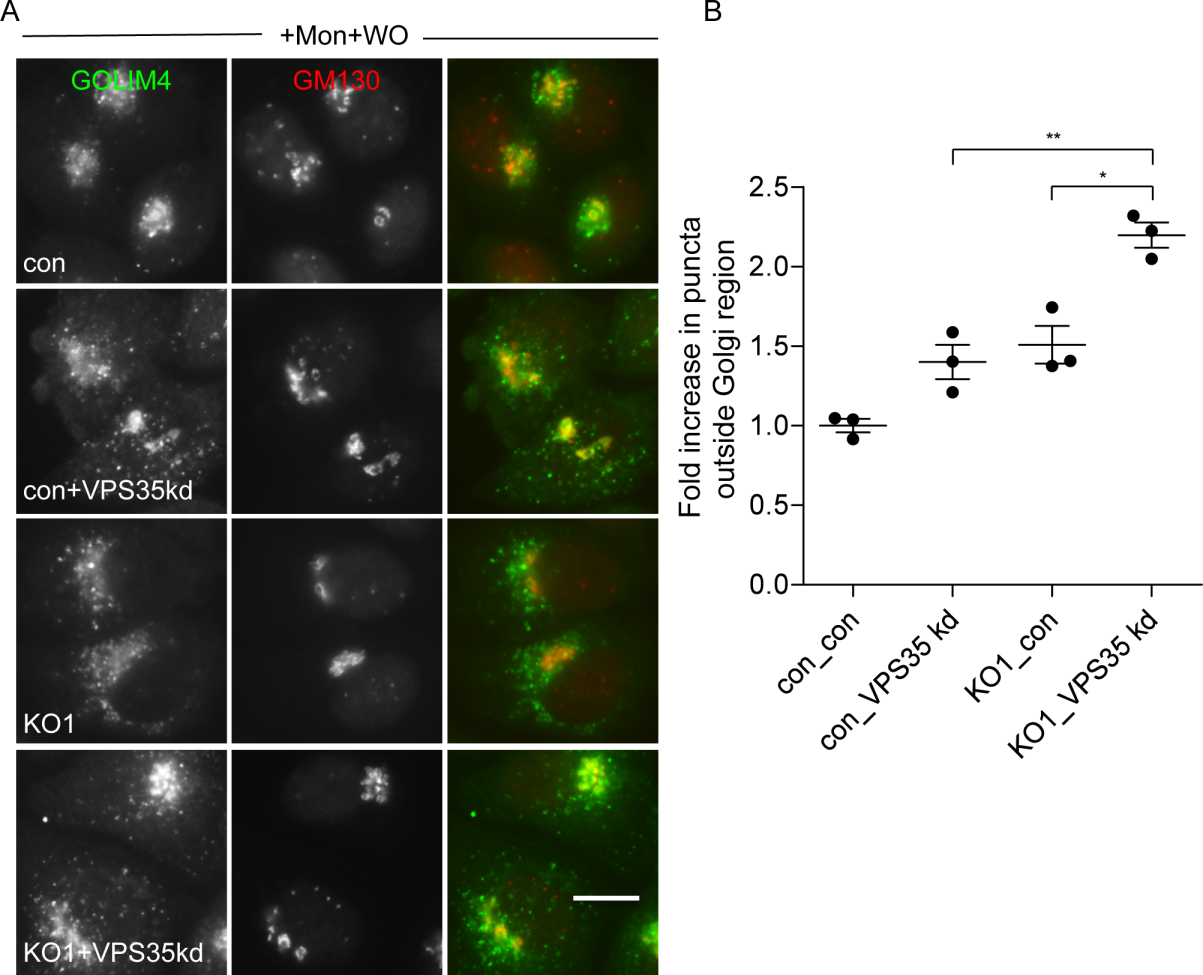
Effect of combined loss of retromer and AP-5 on GOLIM4 retrieval. (A) Immunofluorescence microscopy of cells treated with monensin for 90 min followed by a 2.25-h washout. Individually, the knockdown of retromer or the knockout of AP5Z1 caused a reduction in the retrieval of GOLIM4 back to the Golgi; this was exacerbated when the knockout and knockdown were combined. Scale bar: 20 μm. (B) Quantification of the retrieval defect of GOLIM4 was performed using a CX7 automated microscope and a Colocalisation Bioapplication with an adapted protocol that was originally designed for quantifying CIMPR retrieval (see Fig 3C). The increase in GOLIM4 (Total Object Area) that failed to be retrieved back to the Golgi region was 1.40 ± 0.11 for VPS35 kd, 1.51 ± 0.12 for AP5Z1_KO1 knockout, and 2.20 ± 0.08 for VPS35 kd + AP5Z1 KO. More than 1,500 cells were scored per knockdown condition (3 independent repeats; error bars indicate SEM). The raw data can be found in S4 Data. AP, adaptor protein; CIMPR, cation-independent mannose 6-phosphate receptor; con, control; KO, knockout; Mon, monensin; WO, washout.

We also investigated the effect of knocking down retromer in both wild-type cells and AP-5 knockout cells and then carried out monensin washout experiments. Once again, knocking down retromer alone impaired protein retrieval towards the Golgi, and knocking down retromer in AP-5 knockout cells had an additive effect (Fig 7). Thus, like the CIMPR, GOLIM4 can use both AP-5 and retromer to facilitate endosome-to-Golgi retrieval.

### Binding of SPG15 to CIMPR and sortilin

Adaptor proteins recognise cargo by binding directly to sorting signals in their cytoplasmic tails. The CIMPR has a particularly long cytoplasmic tail (163 residues), with sorting signals for retromer, APs, and GGAs [31,32]. In contrast, the 5 Golgi proteins all have short cytoplasmic tails. For instance, the GOLIM4 tail is only 12 residues long, and deletion studies indicate that it does not contribute to the trafficking of the protein [23]. Thus, although there may be a direct interaction between AP-5/SPG11/SPG15 and the CIMPR, the sorting of the 5 Golgi proteins is likely to be indirect.

**Fig 8:**
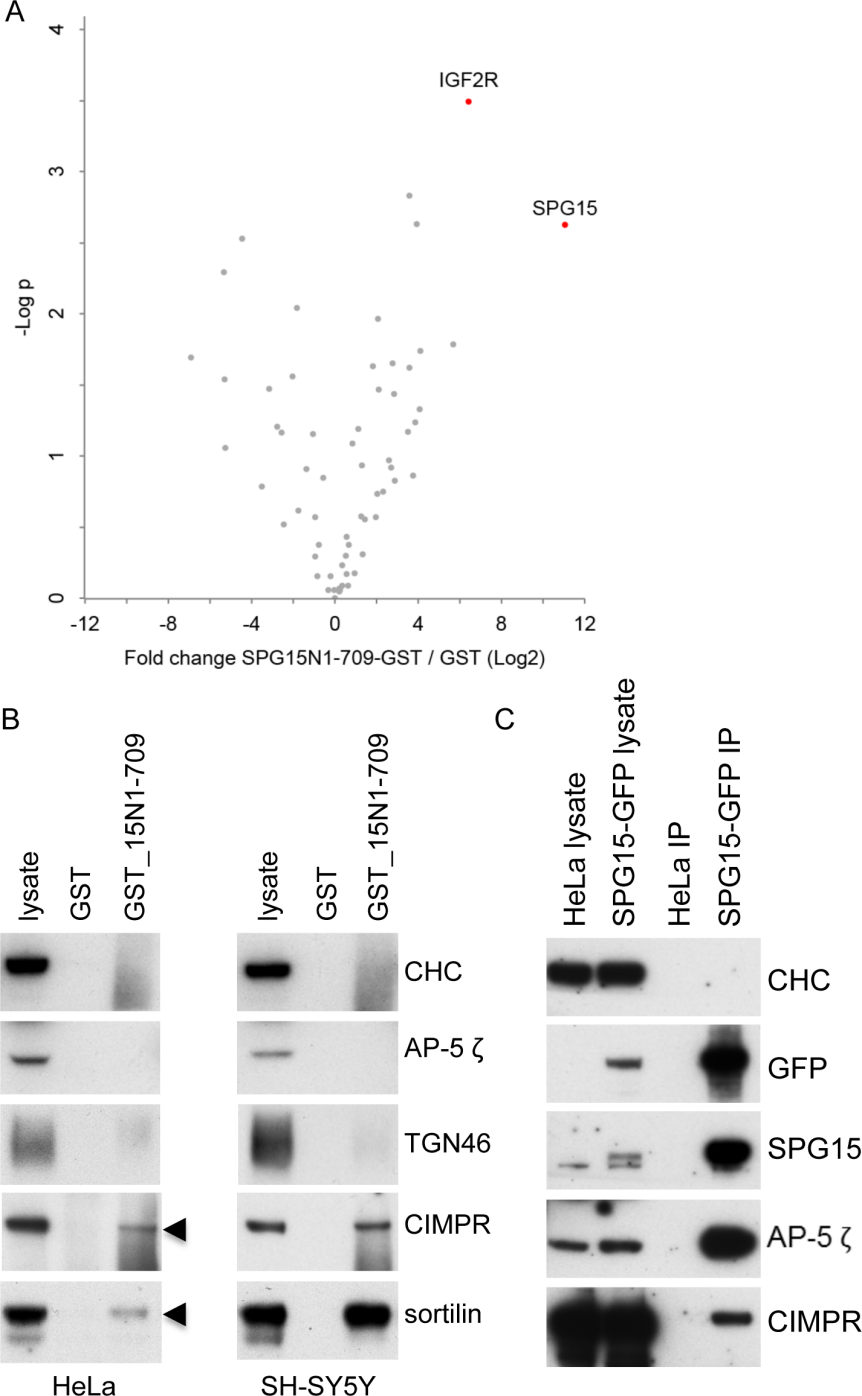
Interactions with CIMPR and sortilin. (A) SPG15/GST pull downs, performed in triplicate. Proteins were analysed by label-free quantification mass spectrometry. The x-axis shows the log_2_-fold change between GST_SPG15N1–709 and GST-only pulldowns; the y-axis shows the −log_10_ *p*-value of significance (2-sided *t* test, *n* = 3 [GST_SPG15N1–709], 4 [GST control]). SPG15 (the bait) and CIMPR/IGF2R are the top hits. Data can be found in S3 Data. (B) Western blots of proteins pulled down either by GST alone or by GST followed by the N-terminal 709 residues of SPG15 (GST_15N1–709), using lysate from either HeLa or SH-SY5Y cells. Using IMAGEJ to quantify bands, we estimate that from the input, GST_15N1–709 pulled down 0.15% of the CIMPR and 0.1% of the sortilin from the HeLa cell lysate, and 0.4% of the CIMPR and 0.6% of the sortilin from the SH-SY5Y lysate. As controls, blots of the lysate and pulldowns were probed with antibodies against CHC, AP-5 ζ, and TGN46. (C) Immunoprecipitations using anti-GFP were carried out on either control HeLa cells or HeLa cells stably expressing SPG15-GFP, and the blots were probed using antibodies, as shown. Both AP-5 ζ and CIMPR are specifically brought down with SPG15-GFP. Using IMAGEJ to quantify bands, we estimate that 65.9% AP-5 ζ and 0.1% CIMPR of input was pulled down by SPG15-GFP, based on 3 repeats. Blots were also probed with an antibody against CHC as a control. AP, adaptor protein; CHC, clathrin heavy chain; CIMPR, cation-independent mannose 6-phosphate receptor; TGN, trans-Golgi network.

To look for potential interactions with cargo proteins, we made several GST fusion proteins from SPG11 and SPG15 as well as from AP-5 subunits and used them to pull down binding partners from cell extracts, which were then identified by mass spectrometry. We found that a construct containing residues 1–709 of SPG15 brought down CIMPR (IGF2R) as one of its top hits from SH-SY5Y neuroblastoma cells (Fig 8A and S3 Data). This interaction was confirmed by western blotting in both HeLa and SH-SY5Y cells (Fig 8B and S2 Fig). We also probed our blots with an antibody against sortilin, which has a similar trafficking itinerary to the CIMPR and some of the same sorting signals in its cytoplasmic tail, even though the tail is shorter (52 residues) and it is much less abundant [4,5]. Again, there was a robust signal in pulldowns from both HeLa and SH-SY5Y cells (Fig 8B). In contrast, TGN46 (used as a control) was not pulled down by the SPG15 construct, nor was the AP-5 ζ subunit, indicating that this domain of SPG15 does not interact with AP-5 (Fig 8B and S2 Fig). We were able to corroborate these interactions in native immunoprecipitations (Fig 8C and S2 Fig).

**Fig 9:**
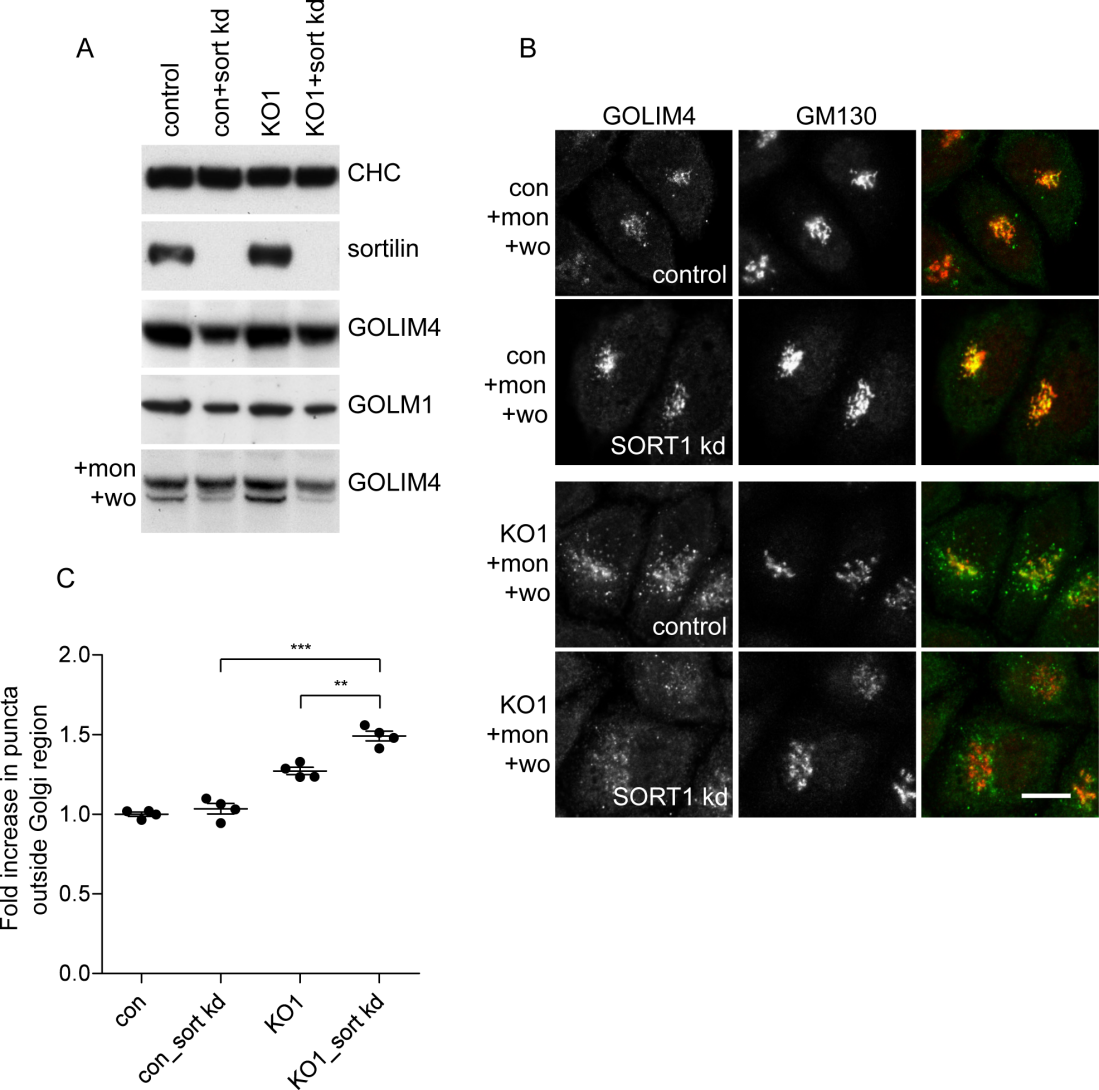
Effect of sortilin knockdown on Golgi protein retrieval. (A) Western blots of wild-type and AP5Z1 knockout cells, either with or without sortilin knockdown, treated with or without monensin for 90 min, followed by a washout for 2.25 h. The knockdown of sortilin caused a reduction of both GOLIM4 and GOLM1 in wild-type and knockout cells (70.7 ± 1.5% GOLIM4 wild type; 78.1 ± 1.6% GOLM1 knockout; 60.3 ± 1.2% GOLM1 wild type; 55.5 ± 6.6% GOLM1 knockout; 3 biological repeats, SEM). (B) Indirect immunofluorescence microscopy of control and AP5Z1 knockout cells, either with or without sortilin knockdown, treated with monensin for 90 min, followed by a 2.25-h washout. The sortilin knockdown alone does not appear to affect the retrieval of GOLIM4 back to the juxtanuclear region. However, knocking down sortilin in AP5Z1 knockout cells exacerbates the retrieval defect. Scale bar: 20 μm. (C) Quantification of the retrieval defect in sortilin knockdown cells, performed using an Arrayscan automated microscope and a Colocalisation Bioapplication with a protocol adapted from the one that was designed for quantifying CIMPR retrieval (Fig 3C). The increase in GOLIM4 (Total Object Area) that failed to be retrieved back to the Golgi region was 1.00 ± 0.02 for control, 1.03 ± 0.04 for control + sortilin knockdown, 1.27 ± 0.03 for AP5Z1 knockout, and 1.49 ± 0.04 for AP5Z1 knockout + sortilin knockdown. More than 1,500 cells were scored per knockdown condition (4 independent repeats; error bars indicate SEM). The raw data can be found in S4 Data. mon, monensin; wo, washout; CHC, clathrin heavy chain; CIMPR, cation-independent mannose 6-phosphate receptor; con, control; KO, knockout; SORT1, sortilin.

Sortilin has been shown to facilitate the trafficking of a number of different cargo proteins, including lysosomal hydrolases, neurotensin, and GLUT4, which bind to its lumenal domain [33–35], while the cytosolic domain of sortilin binds to different types of machinery, including GGAs, AP-1, and retromer [32,36,37]. Interestingly, sortilin was on our list of proteins that were depleted from the vesicle-enriched fraction when AP-5 was knocked out (number 17; the Golgi proteins were 1–5) (S2 Data). This made it a strong candidate for a transmembrane protein that might facilitate the sorting of the AP-5–dependent Golgi proteins. We found that knocking down sortilin caused a reduction in the total amount of both GOLIM4 and GOLM1 (Fig 9A), although their steady-state localisation was unaltered and their behaviour during monensin treatment and washout was indistinguishable from that of control cells (Fig 9B). However, when sortilin was knocked down in AP-5 knockout cells and the cells were then subjected to a monensin washout, there was an increase in non-Golgi-associated GOLIM4, when compared with the AP-5 knockout alone (Fig 9B and Fig 9C). These results support a role for sortilin in the retrieval of GOLIM4 and possibly other Golgi proteins as well. Paradoxically, our findings also suggest that AP-5 may be able to traffic such proteins in a sortilin-independent manner, because if AP-5 acted solely via sortilin, then knocking it out would not exacerbate the phenotype of sortilin knockdown cells.

## Discussion

AP-5 is an ancient and ubiquitous protein complex, but humans and other organisms are able to survive without it. This indicates that even though its loss in humans causes neurological abnormalities, its null phenotype at the cellular level is likely to be subtle. Therefore, instead of trying to predict what AP-5 might be doing, we used 2 large-scale proteomic analyses as unbiased ways of identifying cargo and machinery that either depend upon AP-5 for trafficking or interface somehow with the AP-5 pathway.

The first analysis, dynamic organellar mapping, revealed modest but highly reproducible changes in 2 types of endosomal machinery, the retromer complex, with associated sorting nexins, and the HOPS complex. Although we do not know precisely what these changes mean, they could reflect alterations in endolysosomal dynamics, and/or attempts by the cell to compensate for AP-5 loss. We have previously shown that knocking down AP-5 affects the localisation of retromer, causing it to take on a more clustered appearance [3], and here we show that knocking down retromer also affects the localisation of the AP-5–associated protein SPG15, causing an increase in its membrane association. In addition, we find that knocking out AP-5 impairs retrograde trafficking of the CIMPR towards the TGN and that combining the knockout with a retromer knockdown exacerbates this phenotype. Together, these observations suggest that the CIMPR is a cargo protein for AP-5 and that AP-5 and retromer both contribute to its retrieval.

In the second analysis, we looked for changes in a vesicle-enriched fraction and found that knocking out AP-5 caused several proteins to be depleted, with Golgi membrane proteins among the top hits. Therefore, these 2 approaches are complementary: the first reveals changes in organelles, while the second reveals changes in transport intermediates. Although the steady-state localisation of the Golgi proteins looks normal in AP-5 knockout cells, consistent with our organellar maps, when we shifted their localisation to endosomes with monensin, their retrieval back towards the Golgi was impaired. Again, this phenotype was exacerbated by retromer knockdown.

The Golgi proteins all have short and/or dispensable cytosolic tails, indicating that they do not bind directly to AP-5 or its partners. Thus, we speculated that sortilin, a multipurpose sorting receptor, might act as a bridge between these proteins and the AP-5/SPG11/SPG15 complex. This hypothesis is supported by the presence of sortilin in SPG15 pulldowns and its reduction in vesicle-enriched preparations from AP-5 knockout cells. In addition, while this manuscript was in preparation, Vencat and Linstedt reported that the Mn^2+^-induced exit of GOLIM4 from the Golgi is dependent on sortilin, and they suggested that the lumenal domains of the 2 proteins interact with each other [38]. However, the additive effects of sortilin knockdown and AP-5 knockout indicate that AP-5 can also act in a sortilin-independent manner. Candidates for other proteins that might act as sorting receptors for Golgi proteins include the CIMPR, which also comes down with SPG15 and has a similar tail to sortilin, and other members of the sortilin family, such as SORLA/SORL1 and SORSC1-3.

It will be important to uncover the molecular details of how the AP-5/SPG11/SPG15 complex binds to cargo, especially because there might be similarities to the COPI coat. Phylogenetic analyses indicate that some 2 billion years ago, the ancestor of all the APs, as well as COPI and TSET, was a heterohexamer rather than a heterotetramer, with 2 additional ‘protocoatomer’ subunits consisting of β-propellers, followed by an α-solenoid [39]. These subunits were retained by COPI and TSET but were lost in the AP lineage. However, AP-5 is thought to have been the first complex to branch off after the deep divide between the COPI/TSET family and the AP family, and SPG11 and SPG15 may be descendants of the protocoatomer subunits [39,40]. The ability of SPG15 to bind cargo supports this possibility, because the only known cargo-binding subunits of COPI are associated with the protocoatomers, not the core heterotetramer [41].

Although our pulldown experiments suggest that sortilin and CIMPR can bind to the AP-5/SPG11/SPG15 complex, it is well known that both of these cargo proteins can also bind to other trafficking machinery, including GGAs, AP-1, and retromer. Each of these types of machinery has a somewhat different localisation, enabling them to form transport intermediates from different membranes. For instance, AP-1 localises to a tubular endosomal network [42], while retromer localises to multivesicular bodies [43]. The additive effects of retromer knockdown and AP-5 knockout suggest that AP-5 may act on an alternative retrograde trafficking route, which may in fact be a backup pathway for retromer/sorting nexins and probably for other machinery as well. In other words, most of the time, Golgi-associated proteins would be retrieved before getting to the late endosome, but the few molecules that travel that far would be sent back again by the AP-5/SPG11/SPG15 complex, acting as a last-ditch effort before the terminal lysosome (Fig 9). This type of ‘belt and braces’ scenario, involving interplay between different pathways, is common in membrane traffic. For instance, AP-3 facilitates the trafficking of several lysosomal membrane proteins, but in its absence the proteins can still get to lysosomes by making use of other machinery, albeit less efficiently [44,45]. Using AP-5 as a backup retrograde sorting mechanism would help to explain why AP-5–deficient cells appear to be normal, in most respects, and why AP-5 becomes more important when there are extra demands on retrograde trafficking, such as when Golgi-resident proteins accumulate in endosomes. The gradual buildup of proteins (and probably also lipids) in terminal lysosomes may explain the abnormalities that are seen in fibroblasts from patients with mutations in AP-5, SPG11, or SPG15. Interestingly, previous studies have implicated SPG11 and SPG15 in the reformation of free lysosomes from autolysosomes (i.e., lysosome-autophagosome hybrids) [46,47], a process that is conceptually similar to the retrieval of cargo proteins from endolysosomes during lysosome maturation uncovered by the present study. However, more work is needed to define the relationship between the AP-5 pathway and other pathways more precisely, including the identities of both the donor and the acceptor compartments.

But why does the absence of AP-5 and its partners mainly affect neurons with long axons? This question applies not only to AP-5 but also to all of the other ubiquitously expressed proteins encoded by genes that are mutated in HSP. Part of the answer must be that the exceptional length of these axons puts extra demands on cellular machinery, especially proteins involved in membrane traffic. In addition, a recent study showed that the axons of neurons from humans and mice with mutations in SPG4/spastin develop swellings that are filled with clusters of aberrant lysosomes [12]. These clusters could cause a traffic jam, impeding the progress of other organelles and vesicles up and down the axon [48], eventually causing the axons to degenerate. It will be important to determine whether this is also the case for other HSP subtypes with lysosomal abnormalities, including the subtypes caused by mutations in AP-5, SPG11, and SPG15 as well as the subtype caused by mutations in the SPG8 gene, which encodes a retromer-associated protein, strumpellin [49].

Patients with mutations in AP5Z1 (OMIM #613653), SPG11 (OMIM #610844), or SPG15 (OMIM #270700) have a ‘complicated’ rather than a ‘pure’ form of HSP, with other neurological problems in addition to lower limb spasticity. These include cognitive impairment, thinning of the corpus callosum, and parkinsonism, indicating that the AP-5/SPG11/SPG15 complex contributes to the health of many types of neurons, not just primary motor neurons. Interestingly, a missense mutation in the retromer VPS35 subunit (OMIM #601501) causes an autosomal dominant form of Parkinson disease [50,51], and although the precise molecular mechanism is still unclear, it seems likely that cargo missorting is a key contributing factor in both AP-5/SPG11/SPG15-related HSP and VPS35-related parkinsonism. The identification of manganese-sensitive proteins like GOLIM4 as cargo for both AP-5 and retromer provides a potential clue, because failure to deal with toxic levels of Mn^2+^ has been shown to cause neurodegeneration and parkinsonism [52]. Thus, our findings not only help to clarify the function of an ancient piece of cellular machinery, revealing a novel late-acting retrieval pathway, they also advance our understanding of how endosome/lysosome dysfunction can lead to neurodegenerative disorders, potentially opening up new avenues for the treatment of these diseases.

## Materials and methods

### Antibodies

Antibodies used in this study include in-house antibodies against clathrin [53] and commercial antibodies against AP-5 ζ (Atlas HPA035693), CIMPR (Abcam ab8093), GALNT2 (Abcam ab102650), GOLIM4 (Alexis Biochemicals 804-603-C100), GLG1 (Atlas HPA010815), GOLM1 (Abnova H0005 1280-MO6), GM130 (Abcam ab52649 [rabbit] and BD Transduction Labs 610822 [mouse]), VPS35 (Santa Cruz sc374372), SPG15 (Pro Sci 5023), AP-1 γ (mAb100.3), and sortilin (Abcam ab188586). The rabbit anti-GFP and anti-VPS26 were kind gifts from Matthew Seaman (CIMR, Cambridge, UK). Fluorescently labelled secondary antibodies were purchased from Invitrogen, and HRP-labelled secondary antibodies were purchased from Sigma-Alldrich; western blots were developed using ECL Prime Western Blotting Detection Reagent (GE Healthcare) and quantified using IMAGEJ software.

### CRISPR-Cas9 knockouts

AP5Z1_KO clones were made using CRISPR-Cas9. Guide RNAs targeting AP5Z1 were cloned into pX330 using the ‘simple method protocol’ based on the ‘ELAN’ method described by Cost and Cozzarelli [54], with cotransfection of a G418 selectable plasmid. Cells were maintained in G418 for 3 days, and following cell death, clonal cell lines were established. Although 2 exons (exon 2 and 3) were targeted with 4 different guides, the only one that produced a full knockout was one against exon 3 (ze3g1: CAGAGGGGGACATCTCTCGC), from which 2 clonal knockout lines were established. For AP5Z1_KO1, the sequencing of 26 colonies revealed 2 with a 17–base pair deletion, 2 with a 1–base pair deletion, 3 with a 7–base pair deletion, 3 with 1 base pair substitution plus a 1–base pair deletion, and 16 with a 1–base pair insertion, suggesting the existence of at least 5 alleles. For AP5Z1_KO2 the sequencing of 24 colonies revealed 5 with a 2–base pair deletion, 9 with a 1–base pair deletion, and 10 with a 14–base pair deletion. All mutations are predicted to be deleterious to the expression of ζ protein due to frameshifts.

### Pulldowns and fractionation

The first 709 amino acids of SPG15 were cloned into pGEX4T-1 (GE Healthcare) and sequence verified, to generate the SPG15N1-709 construct. GST alone and SPG15N1-709 were expressed in *Escherichia coli*. For GST pulldowns, HeLa or SH-SY5Y cells were lysed in PBS-T (137 mM NaCl, 2.7 mM KCl, 10 mM Na2HPO4, and 1.76 mM KH2PO4, pH 7.4, adjusted to 0.5% Triton X-100 from a 10% stock) and cleared of debris by centrifugation. Lysates were adjusted to a protein concentration of 2.5 mg/ml, and 50 μg of fusion protein was added as bait for every 4 ml of lysate. The baits and associated proteins were recovered with glutathione Sepharose 4B (GE Healthcare) and eluted with 2.5% (wt/vol) SDS/50 mM Tris, pH 8.0, at 60°C.

For immunoprecipitations, cells were lysed in PBS-T or N (137 mM NaCl, 2.7 mM KCl, 10 mM Na2HPO4, 1.76 mM KH2PO4, and 0.1% [vol/vol] TX100 [Sigma-Aldrich], pH 7.4) and clarified. All samples were precleared by the addition of protein A-Sepharose (GE Healthcare) and then incubated with antibody for 2 h followed by a further hour with protein A–Sepharose. The samples were then washed multiple times in PBS-T/N and immunoprecipitated complexes eluted with sample buffer.

For cytosol and membrane fractions, cells were scraped in PBS and lysed by 8 passages through a 21-gauge needle/5-ml syringe. Nuclei and unbroken cells were removed by centrifugation at 1,000 × g for 5 min, and then membranes were recovered at 100,000 × g for 30 min.

### RNA interference

Knockdowns were performed using the following On-Target Plus SMARTpool siRNA reagents from Dharmacon, with a nontargeting SMARTpool siRNA (D-001810-10) used as a control. The siRNAs for VPS35 were 010894–05 (GAACAUAUUGCUACCAGUA), 010894–06 (GAAAGAGCAUGAGUUGUUA), 010894–07 (GUUGUAAACUGUAGGGAUG), 010894–08 (GAACAAAUUUGGUGCGCCU); for AP5Z1, they were 025284–17 (GGGACUUCGGUGCAGAUUA), 025284–18 (GUUCCUGGGCAGCGUGAAU), 025284–19 (GGAGGUGGCCUUCGAGUAC), 025284–20 (CCACAGACUUCUUCACGGU); and for SORT1, they were 010620–05 (GAGACUAUGUUGUGACCAA), 010620–06 (GAGCUAGGUCCAUGAAUAU), 010620–07 (GAAGGACUAUACCAUAUGG), 010620–08 (GAAUUUGGCAUGGCUAUUG). The custom oligo to knock down VPS26 was a gift from Matthew Seaman [55]. All siRNAs were used at a concentration of 25–50 nM in a 2-hit, 5-day protocol according to manufacturer’s instructions (Dharmacon). Knockdown efficiencies were determined by western blotting and showed >80% depletion for VPS26 and >90% for all other knockdowns (quantified by ImageJ).

### Tissue culture

HeLaM cells [56] and patient fibroblasts [9] were grown in Dulbecco’s Modified Eagle’s Medium (DMEM, Sigma) supplemented with 10% (v/v) foetal calf serum (Sigma), 2 mM L-glutamine, 50 units/ml penicillin, and 50 μg/ml streptomycin. The BAC-transgenic HeLa cell line expressing SPG15-GFP under its own promoter had to be regularly sorted by FACS due to loss of expression. HeLa cells expressing the reporter constructs composed of the cytoplasmic tail of CIMPR (CD8-CIMPR) or sortilin (CD8-sortilin) coupled to the transmembrane and lumenal domain of CD8 were kind gifts from Matthew Seaman [43].

For proteomics, HeLa cells were grown in SILAC medium supplemented with 10% (v/v) dialysed foetal calf serum (10,000 MW cutoff; Invitrogen), penicillin/streptomycin (Sigma), and either “Heavy” amino acids (L-arginine-13C615N4:HCl [50 mg/L] and L-lysine-13C615N2:2HCl [100 mg/L]; Cambridge Isotope Laboratories) or the equivalent “Light” amino acids. Cells were grown for at least 7 days to achieve metabolic labelling, and the average incorporation efficiency was approximately 95%, as determined by mass spectrometry.

### Fluorescence microscopy

For immunofluorescence microscopy, cells were plated onto glass-bottom dishes (MatTek) and treated as indicated with 10 μM monensin or 500 μM manganese. The cells were then fixed with either ice-cold methanol or 3% formaldehyde, permeabilised where necessary with 0.1% saponin and labelled as indicated. The cells were imaged with either a Zeiss Axiovert 200 inverted microscope using a Zeiss Plan Achromat 63× oil immersion objective (NA 1.4), a Hamamatsu OCRA-ER2 camera, and IMPROVISION OPENLAB software or a Zeiss LSM 710 confocal microscope on an inverted AxioImagerZ1 using a Zeiss Plan-Apochromat 63× oil immersion objective (NA1.4) and ZEN Black Software, version 2.3.

To quantify increased fluorescent spot size or retrieval deficits, we used automated high content screening (HCS) microscopes—either an ArrayScan VTI microscope (Cellomics/Thermo Scientific) or its upgrade, a Cell Insight CX7 Microscope (Thermo Scientific). On both instruments, for quantifying fluorescent spot size increases we used a SpotDetector V4 Bioapplication, and for the retrieval assays we used the Colocalisation assay V4 Bioapplication. For the Cellomics we show data from Object Total Area, but subsequently we show Object Count. The ArrayScan VTI consists of a modified Zeiss Axiovert 200M inverted microscope, a Zeiss 40×/0.5NA LD A-Plan objective, a Hamamatsu ORCA-ER camera, and ARRAYSCAN software. The Cell Insight CX7 consists of a custom designed optical platform, Olympus 40×/0.6NA objective, Photometrics X1 camera, and HCS Studio 3.0 software.

To quantify increases in SPG15-GFP fluorescence, cells were plated onto 96-well Perkin Elmer microplates, formaldehyde fixed, and permeabilised, and then the cells were stained with anti-GFP, followed by Alexa Fluor 488-donkey anti-rabbit IgG and then blue whole cell stain (Invitrogen). To quantify retrieval deficits of GOLIM4, cells were plated onto 96-well Perkin Elmer microplates, treated with 10 μM monensin for 90 min, washed, recovered for 2.25 h in fresh media, and then fixed with methanol. The cells were stained with mouse anti-GOLIM4 and rabbit anti-GM130, followed by Alexa Fluor 488-donkey anti-mouse IgG and Alexa Fluor 647 anti-rabbit IgG, followed by blue whole cell stain (Invitrogen). For the CIMPR retrieval assay, cells were plated onto 96-well Perkin Elmer microplates, fed with an antibody that recognises the lumenal domain of CIMPR (Abcam ab8093, at 30 μg/ml) for 15 min at room temperature, and then washed and chased for 60 min. The cells were then fixed with formaldehyde, permabilised with 0.1% saponin, and stained with rabbit anti-mouse and sheep anti-TGN46, followed by Alexa Fluor 647 anti-rabbit IgG and Alexa Fluor 488 anti-sheep IgG and then blue whole cell stain. Controls were included for all steps, with the omission of a single antibody in all combinations.

For both assays, the whole cell stain allowed a mask to be drawn around the cells, and the anti-GM130 or anti-TGN46 allowed a mask to be drawn around the Golgi or TGN, respectively. The amount of fluorescence that was not retrieved back to the Golgi or TGN was then quantified. More than 1,500 cells were scored per knockdown condition, with at least 3 independent repeats. For statistical analysis, data were log transformed prior to analysis by 1-way ANOVA and Tukey-Kramer post hoc test. For pairwise analysis, data were log transformed prior to analysis by paired 2-tailed *t* test (*p < 0.05; **p < 0.01; ***p < 0.001).

### Global proteome determination

For global proteomic analysis of patient fibroblasts, cells were lysed in lysis buffer (2.5% SDS/50 mM Tris-HCl pH = 8) and heated to 90°C. DNA was sheared by passing the lysates through a QIAshredder (Qiagen). Protein concentrations were estimated by BCA assay (Pierce). Proteins were then acetone precipitated and digested prior to analysis by mass spectrometry [5]. Global proteomic analysis of HeLa cells was performed as described by Itzhak et al. [5]. To compare relative protein levels across samples, mass spectrometry data were processed with the MaxLFQ [14] to yield normalised label-free quantification (LFQ) intensities. LFQ intensities were log transformed, and missing data points were imputed from a normal distribution with a downshift of 2.2 and a width of 0.3 standard deviations. Comparison between control and AP-5–deficient cells was performed with a 2-sided *t* test. A permutation-based estimated FDR of 0.12 and an S0 parameter of 0.5 were set to define significance cutoffs (procedure implemented in Perseus software [57]).

### Generation of dynamic organellar maps

Organellar maps were prepared as described in detail in Itzhak et al. [5] from control HeLa cells and the 2 independent AP5Z1 knockout cell lines, in triplicate (9 maps in total). Maps were prepared on 3 separate days, with a complete set of 3 on each occasion (1 control map, 1 map from AP5Z1KO_1, and 1 map from AP5Z1KO_2). In brief, HeLa cells were lysed mechanically, and postnuclear supernatants were subfractionated into 5 fractions by a series of differential centrifugation steps. In parallel, a single membrane fraction was obtained from metabolically ‘heavy’ labelled cells (SILAC method [58]). This fraction served as an internal reference, by spiking it into each of the ‘light’ subfractions. Analysis by mass spectrometry provided a ratio of enrichment/depletion for each protein in each subfraction, relative to the standard. All 5 ratios combined yielded an abundance distribution profile for each protein across the subfractions. Principal component analysis revealed which proteins had similar fractionation profiles (apparent as organellar clusters in S1 Fig).

### Detection of protein profile shifts

To identify proteins that changed subcellular localization in response to AP-5 knockout, we applied our previously described 2-tiered statistical analysis [5,16]. 2,046 proteins were profiled across all 9 maps. Briefly, for each protein, the abundance distribution profile obtained in AP-5 knockout cells was subtracted from the profile obtained in the cognate control map. Thus, for each set of 3 maps, 2 sets of delta profiles were obtained (control–AP5Z1KO_1 and control–AP5Z1KO_2). First, all delta profile sets were subjected to a robust multivariate outlier test to identify proteins with delta profiles that are significantly above experimental scatter. Second, the reproducibility of observed delta profiles across repeats was determined as the Pearson correlation (replicates 1vs2, 1vs3, and 2vs3). Hence, we obtained 2 times 3 *p*-values for movement, and 2 times 3 profile correlations. We previously described in detail how to analyse such data for triplicate repeats of a control versus ‘treatment’ experiment (i.e., 3 *p*-values and 3 correlations [5,16]. In the present study, we modified the analysis to accommodate the second set of ‘treatment’ samples (i.e., 2 AP-5 knockout cell lines). Because the shifts induced by AP5 KO are very subtle, we used our intermediate stringency scoring for higher sensitivity [5]. For each of the 2 AP5Z1 knockout clones, we selected the median observed *p*-value for movement and the median observed correlation. To combine the results for the 2 AP-5 knockout clones, we then selected the higher of the 2 median *p*-values (i.e., the less significant one) and the lower correlation. This correlation corresponds to the protein’s R score. The *p*-value of movement was then squared (because a *p*-value at least as small as this was observed in 2 independent experiments) and corrected for multiple hypothesis testing, using the Benjamini-Hochberg method. The −log_10_ of the corrected *p*-value corresponded to the protein’s M score. As an additional filter, we also scored the correlation of the delta profiles obtained with the 2 AP5Z1 knockout clones (replicates 1vs2, 1vs3, and 2vs3) and selected the median clone correlation. Only proteins with a clone correlation >0.75 (i.e., similar movement in both AP-5 knockout clones) were considered as candidate movers. We then used our previously published HeLa maps [5] (3 pairs of untreated maps with no genuine protein shifts expected) to control the FDR. As above, we calculated M and R scores from median correlations and *p*-values of movement. The estimated FDR for a given set of M and R score cutoffs corresponds to the number of hits obtained with the mock experiment data, divided by the number of hits obtained with the AP-5 experiment data, scaled by the relative sizes of the datasets. The cutoffs chosen in Fig 1C (M > 1.5, R > 0.5) correspond to an estimated FDR of 23%. Please note that the actual FDR is probably lower than this estimated FDR, because the mock data lack the additional cell line and the clonal correlation filter.

### Vesicle-enriched fraction

Control cells were grown in SILAC Heavy medium and AP5Z1_KO1 (or KO2) cells were grown in SILAC Light medium, mixed, and a vesicle-enriched fraction was isolated. Five confluent 10-cm dishes were scraped into 3.5 ml of buffer A (0.1 M MES, pH 6.5 [adjusted with NaOH], 0.2 mM EGTA, and 0.5 mM MgCl_2_). Cells were homogenised with a motorised Potter-Elvehjem homogeniser (16 strokes) and centrifuged at 4,100 g for 32 min. Supernatants were treated with ribonuclease A at 50 μg/ml for 60 min. Partially digested ribosomes were pelleted by centrifugation (4,100 g for 3 min) and discarded. Membranes were pelleted by centrifugation at 55,000 rpm (209,900 g RCFmax) for 40 min in an MLA-80 rotor (Beckman Coulter). Membranes were resuspended in 300 μl buffer A using a 1 ml Dounce homogeniser and mixed with an equal volume of FS buffer (12.5% [wt/vol] Ficoll and 12.5% [wt/vol] sucrose, in buffer A). Samples were spun at 20,000 rpm (21,700 g RCFmax) for 34 min to pellet the larger particles (pellet discarded). Supernatants were diluted with 4 volumes of buffer A and centrifuged at 40,000 rpm (86,700 g RCFmax) in a TLA-110 rotor for 30 min to obtain the vesicle-enriched fraction (pellet). All preparations were performed at 4°C.

A maximum total of 50 μg protein was loaded onto precast gels (NuPAGE 4%–12% Bis Tris Gels; Invitrogen) and run so that the sample separated into a 5-cm strip. The gel was then washed, stained with Coomassie blue, and cut into 10 slices. Proteins were reduced, alkylated with iodoacetamide (A3221, Sigma), and in-gel digested with trypsin and the sample analysed by LC-MSMS using a Q-Exactive mass spectrometer (Q-Exactive [59])

### Mass spectrometric analysis of organellar map samples and cell lysates

Protein samples were prepared for mass spectrometry essentially as described [5]. Briefly, following tryptic digest, peptide cleanup and/or fractionation was performed on SDB-RPS Stage tips. Peptides were then loaded onto a 50-cm column (75-μm inner diameter, packed in-house with 1.8 μm C18 particles) (Dr. Maisch GmbH, Germany) and separated with an EASY-nLC 1000 (Thermo Fisher Scientific, Germany). For organellar map samples, peptide analysis was performed on a Q Exactive HF Hybrid Quadrupole-Orbitrap mass spectrometer (Thermo Fisher Scientific, Germany), without additional peptide fractionation (150 min HPLC gradient/sample). The first replicate of the patient fibroblast full proteomes was analysed on an Exactive mass spectrometer (Thermo Fisher Scientific, Germany), following peptide fractionation by SAX (as in [60]; 6 fractions/sample; 240 min HPLC gradients). The second replicate was analysed on a Q Exactive HF mass spectrometer, following peptide fractionation by SDB-RPS (as in [61]; 3 fractions/sample; 150 min HPLC gradients). Raw files were processed with MaxQuant [62] using the human reference database (SwissProt canonical and isoforms data) downloaded from UniProt.

### Data analysis for vesicle-enriched fractions

For the AP5Z1 KO, datasets were produced of 2 independent biological repeats of control (Heavy SILAC) and both AP5Z1_KO1 (Light SILAC) and AP5Z1_KO2 (Light SILAC). The raw data files were processed using MaxQuant. The primary output for each SILAC comparison of vesicle-enriched fraction was a list of identified proteins, a ratio of relative abundance (Ratio H/L), a measure of the variability within each mass spec run (Ratio H/L variability [%]), and the number of quantification events (Ratio H/L count).

The raw data identified 2,500 proteins across the 4 different datasets. The data were then formatted as follows. Reverse hits, proteins only identified by site, common contaminants, and proteins with no gene names were removed. The ratios of H/L were then determined and linearly normalised based on total intensities, assuming equal protein quantities in both Heavy and Light samples. Because control cells were labelled with Heavy SILAC and knockout cells with Light SILAC, an H/L ratio >1 represents depletion from the vesicle-enriched fraction. The data were then filtered to remove proteins that were identified in <4 experiments, those with variability within each experiment (>40% average variability over 4 experiments), those with large variability between paired repeats (SD >1), or those with low counts (<4 average over 4 experiments). This left a final list of about 700 proteins, which were then ranked in order of greatest depletion from the vesicle-enriched fraction (S2 Data).

### Software for bioinformatic analysis of proteomic data

Proteomic data transformation, filtering, and statistical analysis were performed in Perseus software [57], Prism 6 (GraphPad Software), and Microsoft Excel. Principal component analysis was performed in SIMCA 14 (Umetrics/MKS).

## Supporting information

S1 Fig (A–C) Organellar maps from HeLa control (A) and AP-5 knockout cells (B, AP5Z1_KO1; C, AP5Z1_KO2), visualised by PCA. Each scatter point corresponds to a protein; proximity of proteins suggests similar fractionation behaviour and hence similar organellar association. Established marker proteins of various compartments are shown in colour. Proteins that undergo a significant shift upon AP-5 ablation are indicated with white centres. Each map combines the profiling data from 3 independent replicates (i.e., 15 data points per protein). Plots for all maps were generated in a single PCA to ensure maximum comparability (as in [15]). Projections along the first (x-axis) and third (y-axis) principal components provide the optimal visual separation of clusters; together, they account for >75% of the variability in the data. (D, E) Close-up on the endosomal cluster, where most significant protein movements occur. The control map is shown, and the shifts of retromer subunits (VPS29, VPS35, SNX2, SNX3, SNX5, SNX27), HOPS subunits (VPS16, VPS18, VPS33a, VPS39), and cation-independent mannose 6-phosphate receptor (IGF2R) in the 2 AP-5 knockout maps are indicated with arrows. These proteins undergo strikingly similar movements within the endosomal cluster, moving towards the lysosomal cluster (salmon-coloured dots). Nonmarker proteins (small grey dots) shown in the parent control map have been removed from the close-ups to enhance clarity. AP, adaptor protein; KO, knockout; PCA, principal component analysis.
(PDF)

S2 Fig Interactions between AP-5/SPG11/SPG15 and cargo investigated by immunoprecipitation. (A) Immunoprecipitations using anti-GFP were carried out on either control HeLa cells or HeLa cells stably expressing SPG15-GFP, and the blots were probed using antibodies, as shown. Both AP-5 ζ and CIMPR are specifically brought down with SPG15-GFP. Using IMAGEJ to quantify bands, we estimate that 65.9% AP-5 ζ and 0.1% CIMPR of input was pulled down by SPG15-GFP, based on 3 repeats. Blots were also probed with an antibody against CHC as a control. (B) Immunoprecipitations using anti-CD8 were carried out on either control HeLa cells or HeLa cells expressing CD8-CIMPR or CD8-sortilin, and the blots were probed using antibodies, as shown. AP-1 γ, SPG15, and AP-5 ζ are specifically brought down with CD8-CIMPR and CD8-sortilin. Using IMAGEJ to quantify bands, we estimate that 0.1% AP-1 γ, 0.15% SPG15, and 0.08% AP-5 ζ of input was pulled down by CD8-CIMPR, and 0.05% AP-1 γ, 0.15% SPG15, and 0.23% AP-5 ζ of input was pulled down by CD8-sortilin, based on 3 repeats. Blots were also probed with an antibody against CHC as a control. AP, adaptor protein; CHC, clathrin heavy chain; CIMPR, cation-independent mannose 6-phosphate receptor; SPG, spastic paraplegia gene.
(PDF)

S1 Data Data from full proteome analyses and organellar mapping. This table also contains the complete results of the dynamic organellar maps MR plot analysis and the GO-term enrichment analysis of proteins shifting in response to AP-5 ablation. AP, adaptor protein.
(XLSX)

S2 Data Proteomic analysis of a vesicle-enriched preparation from SILAC-labelled AP5Z1 knockout cells lines. The fold change (control:KO) was calculated for each protein for both knockout lines (AP5Z1_KO1 and AP5Z1_KO2) in 2 biological repeats and the results averaged. Proteins were ranked from the highest to lowest ratio. The top 5 hits are all Golgi-associated proteins. A number of other potentially interesting proteins are highlighted by asterisks. These include several lysosomal hydrolases that are mutated in lysosomal storage diseases (GBA, HEXA, GNS), lipases (PLD1, DAGLB), proteins associated with either endosomes (TBC1D5, PIK3R4, SORT1) or lysosomal positioning (BORCS6, ARL8A, ARL8B), and proteins associated with neuronal function (ATXN10, SYNGR3). KO, knockout; SILAC, stable isotope labelling of amino acids in cell culture; SORT1, sortilin.
(XLSX)

S3 Data Data from the SPG15/GST pulldown experiments used to generate Fig 8A. Note that SPG15 was identified by its alternative name, ZFYVE26. SPG, spastic paraplegia gene.
(XLSX)

S4 Data Raw data from the automated microscopy experiments shown in Figs 2, 3, 4, 6, 7 and 9. (XLSX)

## Abbreviations

Ab: antibody
AP: adaptor protein
CCV: clathrin-coated vesicle
CHC: clathrin heavy chain
CIMPR: cation-independent mannose 6-phosphate receptor
con: control
CYT: cytosol
DMEM: Dulbecco’s Modified Eagle’s Medium
HCS: high content screening
HSP: hereditary spastic paraplegia
KO: knockout
LFQ: label-free quantification
M: magnitude
MEM: membrane
Mn^2+^: manganese
Mon: monensin
PCA: principal component analysis
PNS: postnuclear supernatant
R: reproducibility
SILAC: stable isotope labelling of amino acids in cell culture
siRNA: small interfering RNA
SORT1: sortilin
SPG: spastic paraplegia gene
TGN: trans-Golgi network
WCS: whole cell stain
WO: washout
wt: wild-type

## References

[1] Robinson MS (2015) Forty years of clathrin-coated vesicles. Traffic 16: 1210–1238. doi: 10.1111/tra.12335 26403691 10.1111/tra.12335

[2] Hirst J, Barlow LD, Francisco GC, Sahlender DA, Seaman MNJ, et al (2011) The fifth adaptor protein complex. PLoS Biol 9: e1001170 doi: 10.1371/journal.pbio.1001170 22022230 10.1371/journal.pbio.1001170 PMC3191125

[3] Hirst J, Borner GHH, Edgar J, Hein MY, Mann M, et al (2013) Interaction between AP-5 and the hereditary spastic paraplegia proteins SPG11 and SPG15. Mol Biol Cell 24: 2558–2569. doi: 10.1091/mbc.E13-03-0170 23825025 10.1091/mbc.E13-03-0170 PMC3744948

[4] Hein MY, Hubner NC, Poser I, Cox J, Nagaraj N, et al (2015) A human interactome in three quantitative dimensions organized by stoichiometries and abundances. Cell 163: 712–723. doi: 10.1016/j.cell.2015.09.053 26496610 10.1016/j.cell.2015.09.053

[5] Itzhak DN, Tyanova S, Cox J, Borner GHH (2016) Global, quantitative and dynamic mapping of protein subcellular localization. eLife 5: e16950 doi: 10.7554/eLife.16950 27278775 10.7554/eLife.16950 PMC4959882

[6] Hirst J, Irving C, Borner GHH (2013) Adaptor protein complexes AP-4 and AP-5: new players in endosomal trafficking and progressive spastic paraplegia. Traffic 14: 153–164. doi: 10.1111/tra.12028 23167973 10.1111/tra.12028

[7] Słabicki M, Theis M, Krastev DB, Samsonov S, Mundwiller E, et al (2010) A genome-scale DNA repair RNAi screen identifies SPG48 as a novel gene associated with hereditary spastic paraplegia. PLoS Biol 8: e1000408 doi: 10.1371/journal.pbio.1000408 20613862 10.1371/journal.pbio.1000408 PMC2893954

[8] Blackstone C, O'Kane CJ, Reid E (2011) Hereditary spastic paraplegias: membrane traffic and the motor pathway. Nat Rev Neurosci 12: 31–42. doi: 10.1038/nrn2946 21139634 10.1038/nrn2946 PMC5584382

[9] Hirst J, Edgar JR, Esteves T, Darios F, Madeo M, et al (2015) Loss of AP-5 results in accumulation of aberrant endolysosomes: defining a new type of lysosomal storage disease. Hum Mol Genet 24: 4984–4896. doi: 10.1093/hmg/ddv220 26085577 10.1093/hmg/ddv220 PMC4527494

[10] Khundadze M, Kollmann K, Koch N, Biskup C, Nietzsche S, et al (2013) A hereditary spastic paraplegia mouse model supports a role of ZFYVE26/SPASTIZIN for the endolysosomal system. PLoS Genet 9: e1003988 doi: 10.1371/journal.pgen.1003988 24367272 10.1371/journal.pgen.1003988 PMC3868532

[11] Renvoisé B, Chang J, Singh R, Yonekawa S, FitzGibbon EJ, et al (2014) Lysosomal abnormalities in hereditary spastic paraplegia types SPG15 and SPG11. Ann Clin Transl Neurol 1: 379–389. doi: 10.1002/acn3.64 24999486 10.1002/acn3.64 PMC4078876

[12] Allison R, Edgar JR, Pearson G, Rizo T, Newton T, et al (2017) Defects in ER-endosome contacts impact lysosome function in hereditary spastic paraplegia. J Cell Biol 216: 1337–1355. doi: 10.1083/jcb.201609033 28389476 10.1083/jcb.201609033 PMC5412567

[13] Traub LM, Bonifacino JS (2013) Cargo recognition in clathrin-mediated endocytosis. Cold Spring Harb Perspect Biol 5: a016790 doi: 10.1101/cshperspect.a016790 24186068 10.1101/cshperspect.a016790 PMC3809577

[14] Cox J, Hein MY, Luber CA, Paron I, Nagaraj N, et al (2014) Accurate proteome-wide label-free quantification by delayed normalization and maximal peptide ratio extraction, termed MaxLFQ. Mol Cell Proteomics 13: 2513–2526. doi: 10.1074/mcp.M113.031591 24942700 10.1074/mcp.M113.031591 PMC4159666

[15] Hirst J, Madeo M, Smets K, Edgar JR, Schols L, et al (2016) Complicated spastic paraplegia in patients with AP5Z1 mutations (SPG48). Neurol Genet 2: e98 doi: 10.1212/NXG.0000000000000098 27606357 10.1212/NXG.0000000000000098 PMC5001803

[16] Itzhak DN, Colin Davies C, Tyanova S, Mishra A, Williamson J, et al (2017) A mass-spectrometry based approach for mapping protein subcellular localization reveals the spatial proteome of mouse primary neurons. Cell Rep In press. 10.1016/j.celrep.2017.08.063 PMC5775508 28903049

[17] Breusegem SY, Seaman MNJ (2014) Genome-wide RNAi screen reveals a role for multipass membrane proteins in endosome-to-Golgi retrieval. Cell Rep: 1931–1945. doi: 10.1016/j.celrep.2014.10.053 25464851 10.1016/j.celrep.2014.10.053 PMC4542293

[18] Kamiyama S, Suda T, Ueda R, Suzuki M, Okubo R, et al (2003) Molecular cloning and identification of 3'-phosphoadenosine 5'-phosphosulfate transporter. J Biol Chem 278: 25958–25963. doi: 10.1074/jbc.M302439200 12716889 10.1074/jbc.M302439200

[19] Kladney RD, Bulla GA, Guo L, Mason AL, Tollefson AE, et al (2000) GP73, a novel Golgi-localized protein upregulated by viral infection. Gene 249: 53–65. 10831838 10.1016/S0378-1119(00)00136-0 PMC7127640

[20] Linstedt AD, Mehta A, Suhan J, Reggio H, Hauri HP (1997) Sequence and overexpression of GPP130/GIMPc: evidence for saturable pH-sensitive targeting of a type II early Golgi membrane protein. Mol Biol Cell 8: 1073–1087. 9201717 10.1091/mbc.8.6.1073 PMC305715

[21] Mourelatos Z, Gonatas JO, Nycum LM, Gonatas NK, Biegel JA (1995) Assignment of the GLG1 gene for MGF-160, a fibroblast growth factor and E-selectin binding membrane sialoglycoprotein of the Golgi apparatus, to chromosome 16q22-q23 by fluorescence in situ hybridization. Genomics 28: 354–355. 8530051 10.1006/geno.1995.1156

[22] White T, Bennett EP, Takio K, Sørensen T, Bonding N, et al (1995) Purification and cDNA cloning of a human UDP-N-acetyl-alpha-D-galactosamine:polypeptide N-acetylgalactosaminyltransferase. J Biol Chem 270: 24156–24165. 7592619 10.1074/jbc.270.41.24156

[23] Bachert C, Lee TH, Linstedt AD (2001) Lumenal endosomal and Golgi-retrieval determinants involved in pH-sensitive targeting of an early Golgi protein. Mol Biol Cell 12: 3152–3160. 11598199 10.1091/mbc.12.10.3152 PMC60163

[24] Puri S, Bachert C, Fimmel CJ, Linstedt AD (2002) Cycling of early Golgi proteins via the cell surface and endosomes upon lumenal pH disruption. Traffic 3: 641–653. 12191016 10.1034/j.1600-0854.2002.30906.x

[25] Natarajan R, Linstedt AD (2004) A cycling cis-Golgi protein mediates endosome-to-Golgi traffic. Mol Biol Cell 15: 4798–4806. doi: 10.1091/mbc.E04-05-0366 15331763 10.1091/mbc.E04-05-0366 PMC524728

[26] Mukhopadhyay S, Bachert C, Smith DR, Linstedt AD (2010) Manganese-induced trafficking and turnover of the cis-Golgi glycoprotein GPP130. Mol Biol Cell 21: 1282–1292. doi: 10.1091/mbc.E09-11-0985 20130081 10.1091/mbc.E09-11-0985 PMC2847531

[27] Robinson MS, Sahlender DA, Foster SD (2010) Rapid inactivation of proteins by rapamycin-induced rerouting to mitochondria. Dev Cell 18: 324–331. doi: 10.1016/j.devcel.2009.12.015 20159602 10.1016/j.devcel.2009.12.015 PMC2845799

[28] Hirst J, Borner GHH, Antrobus R, Peden AA, Hodson NA, et al (2012) Distinct and overlapping roles for AP-1 and GGAs revealed by the "knocksideways" system. Curr Biol 22: 1711–1716. doi: 10.1016/j.cub.2012.07.012 22902756 10.1016/j.cub.2012.07.012 PMC3485558

[29] Tewari R, Bachert C, Linstedt AD (2015) Induced oligomerization targets Golgi proteins for degradation in lysosomes. Mol Biol Cell 26: 4427–4437. doi: 10.1091/mbc.E15-04-0207 26446839 10.1091/mbc.E15-04-0207 PMC4666137

[30] Chapman RE, Munro S (1994) Retrieval of TGN proteins from the cell surface requires endosomal acidification. EMBO J 13: 2305–2312. 8194522 10.1002/j.1460-2075.1994.tb06514.x PMC395094

[31] Bonifacino JS, Traub LM (2003) Signals for sorting of transmembrane proteins to endosomes and lysosomes. Ann Rev Biochem 72: 395–447. doi: 10.1146/annurev.biochem.72.121801.161800 12651740 10.1146/annurev.biochem.72.121801.161800

[32] Seaman MNJ (2007) Identification of a novel conserved sorting motif required for retromer-mediated endosome-to-TGN retrieval. J Cell Sci 120: 2378–2389. doi: 10.1242/jcs.009654 17606993 10.1242/jcs.009654

[33] Bogan JS, Kandror KV (2010) Biogenesis and regulation of insulin-responsive vesicles containing GLUT4. Curr Opin Cell Biol 22: 506–512. doi: 10.1016/j.ceb.2010.03.012 20417083 10.1016/j.ceb.2010.03.012 PMC2910140

[34] Canuel M, Libin Y, Morales CR (2009) The interactomics of sortilin: an ancient lysosomal receptor evolving new functions. Histol Histopathol 24: 481–492. doi: 10.14670/HH-24.481 19224451 10.14670/HH-24.481

[35] Nykjaer A, Willnow TE (2012) Sortilin: a receptor to regulate neuronal viability and function. Trends Neurosci 35: 261–270. doi: 10.1016/j.tins.2012.01.003 22341525 10.1016/j.tins.2012.01.003

[36] Braulke T, Bonifacino JS (2009) Sorting of lysosomal proteins. Biochim Biophys Acta 1793: 605–614. doi: 10.1016/j.bbamcr.2008.10.016 19046998 10.1016/j.bbamcr.2008.10.016

[37] Nielsen MS, Madsen P, Christensen EI, Nykjaer A, Gliemann J, et al (2001) The sortilin cytoplasmic tail conveys Golgi-endosome transport and binds the VHS domain of the GGA2 sorting protein. EMBO J In press. 10.1093/emboj/20.9.2180 PMC125444 11331584

[38] Venkat S, Linstedt AD (2017) Manganese-induced trafficking and turnover of GPP130 is mediated by sortilin. Mol Biol Cell E17-05-0326. 10.1091/mbc.E17-05-0326 PMC5597328 28768823

[39] Hirst J, Schlacht A, Norcott JP, Traynor D, Bloomfield G, et al (2014) Characterization of TSET, an ancient and widespread membrane trafficking complex. eLife 3: e02866 doi: 10.7554/eLife.02866 24867644 10.7554/eLife.02866 PMC4031984

[40] Dacks JB, Robinson MS (2017) Outerwear through the ages: evolutionary cell biology of vesicle coats. Curr Opin Cell Biol 47: 108–116. doi: 10.1016/j.ceb.2017.04.001 28622586 10.1016/j.ceb.2017.04.001

[41] Jackson LP, Lewis MJ, Kent HM, Edeling MA, Evans PR, et al (2012) Molecular basis for recognition of dilysine trafficking motifs by COPI. Dev Cell 11: 1255–1262. 10.1016/j.devcel.2012.10.017 PMC3521961 23177648

[42] Peden AA, Ooeschot V, Hesser BA, Austin CD, Scheller RH, et al (2003) Localization of the AP-3 adaptor complex defines a novel endosomal exit site for lysosomal membrane proteins. J Cell Biol 164: 1065–1076. 10.1083/jcb.200311064 PMC2172074 15051738

[43] Seaman MNJ (2004) Cargo-selective endosomal sorting for retrieval to the Golgi requires retromer. J Cell Biol 165: 111–122. doi: 10.1083/jcb.200312034 15078902 10.1083/jcb.200312034 PMC2172078

[44] Dell'Angelica EC, Shotelersuk V, Aguilar RC, Gahl WA, Bonifacino JS (1999) Altered trafficking of lysosomal proteins in Hermansky-Pudlak syndrome due to mutations in the beta 3A subunit of the AP-3 adaptor. Mol Cell 3: 11–21. 10024875 10.1016/s1097-2765(00)80170-7

[45] Peden AA, Rudge RE, Lui WW, Robinson MS (2002) Assembly and function of AP-3 complexes in cells expressing mutant subunits. J Cell Biol 156: 327–336. doi: 10.1083/jcb.200107140 11807095 10.1083/jcb.200107140 PMC2199225

[46] Chang J, Lee S, Blackstone C (2014) Spastic paraplegia proteins spastizin and spatacsin mediate autophagic lysosome reformation. J Clin Invest 124: 5249–5262. doi: 10.1172/JCI77598 25365221 10.1172/JCI77598 PMC4348974

[47] Varga RE, Khundadze M, Damme M, Nietzsche S, Hoffmann B, et al (2015) In vivo evidence for lysosome depletion and impaired autophagic clearance in Hereditary Spastic Paraplegia Type SPG11. PLoS Genet 11: e1005454 doi: 10.1371/journal.pgen.1005454 26284655 10.1371/journal.pgen.1005454 PMC4540459

[48] Tsukita S, Ishikawa H (1980) The movement of membranous organelles in axons. Electron microscopic identification of anterogradely and retrogradely transported organelles. J Cell Biol 84: 513–530. 6153657 10.1083/jcb.84.3.513 PMC2110575

[49] Freeman C, Seaman MNJ, Reid E (2013) The hereditary spastic paraplegia protein strumpellin: characterisation in neurons and of the effect of disease mutations on WASH complex assembly and function. Biochim Biophys Acta 1832: 160–173. doi: 10.1016/j.bbadis.2012.10.011 23085491 10.1016/j.bbadis.2012.10.011 PMC3714738

[50] Vilariño-Güell C, Wider C, Ross OA, Dachsel JC, Kachergus JM, et al (2011) VPS35 mutations in Parkinson disease. Am J Hum Genet 89: 162–167. doi: 10.1016/j.ajhg.2011.06.001 21763482 10.1016/j.ajhg.2011.06.001 PMC3135796

[51] Zimprich A, Benet-Pagès A, Struhal W, Graf E, Eck SH, et al (2011) A mutation in VPS35, encoding a subunit of the retromer complex, causes late-onset Parkinson disease. Am J Hum Genet 89: 168–175. doi: 10.1016/j.ajhg.2011.06.008 21763483 10.1016/j.ajhg.2011.06.008 PMC3135812

[52] Aschner M, Erikson KM, Herrero Hernández E, Tjalkens R (2009) Manganese and its role in Parkinson's disease: from transport to neuropathology. Neuromolecular Med 11: 252–266. doi: 10.1007/s12017-009-8083-0 19657747 10.1007/s12017-009-8083-0 PMC4613768

[53] Simpson F, Bright NA, West MA, Newman LS, Darnell RB, et al (1996) A novel adaptor-related protein complex. J Cell Biol 133: 749–760. 8666661 10.1083/jcb.133.4.749 PMC2120832

[54] Cost GJ, Cozzarelli NR (2007) Directed assembly of DNA molecules via simultaneous ligation and digestion. Biotechniques 42: 86–89. 10.2144/000112283 17269489

[55] Gokool S, Tattersall D, Seaman MNJ (2007) EHD1 interacts with retromer to stabilize SNX1 tubules and facilitate endosome-to-Golgi retrieval. Traffic 8: 1873–1886. doi: 10.1111/j.1600-0854.2007.00652.x 17868075 10.1111/j.1600-0854.2007.00652.x

[56] Tiwari RK, Kusari J, Sen GC (1987) Functional equivalents of interferon-mediated signals needed for induction of an mRNA can be generated by double-stranded RNA and growth factors. EMBO J 6: 3373–3378. 2828026 10.1002/j.1460-2075.1987.tb02659.x PMC553793

[57] Tyanova S, Temu T, Sinitcyn P, Carlson A, Hein MY, et al (2016) The Perseus computational platform for comprehensive analysis of (prote)omics data. Nat Methods 13: 731–740. doi: 10.1038/nmeth.3901 27348712 10.1038/nmeth.3901

[58] Ong SE, Blagoev B, Kratchmarova I, Kristensen DB, Steen H, et al (2002) Stable isotope labeling by amino acids in cell culture, SILAC, as a simple and accurate approach to expression proteomics. Mol Cell Proteomics 1: 376–386. 12118079 10.1074/mcp.m200025-mcp200

[59] Borner GHH, Antrobus R, Hirst J, Bhumbra GS, Kozik P, et al (2012) Multivariate proteomic profiling identifies novel accessory proteins of coated vesicles. J Cell Biol 197: 141–160. doi: 10.1083/jcb.201111049 22472443 10.1083/jcb.201111049 PMC3317806

[60] Wiśniewski JR, Zougman A, Nagaraj N, Mann M (2009) Universal sample preparation method for proteome analysis. Nat Methods 6: 359–362. doi: 10.1038/nmeth.1322 19377485 10.1038/nmeth.1322

[61] Kulak NA, Pichler G, Paron I, Nagaraj N, Mann M (2014) Minimal, encapsulated proteomic-sample processing applied to copy-number estimation in eukaryotic cells. Nat Methods 11: 319–324. doi: 10.1038/nmeth.2834 24487582 10.1038/nmeth.2834

[62] Cox J, Mann M (2008) MaxQuant enables high peptide identification rates, individualized p.p.b.-range mass accuracies and proteome-wide protein quantification. Nat Biotechnol 26: 1367–1372. doi: 10.1038/nbt.1511 19029910 10.1038/nbt.1511

