# Supplementary material for: Role of the AP-5 adaptor protein complex in late endosome-to-Golgi retrieval
Source: PLoS Biol. 2018 Jan 30;16(1):e2004411. doi: 10.1371/journal.pbio.2004411 (PMC5806898; doi:10.1371/journal.pbio.2004411)
Supplement: S1 Fig — (A–C) Organellar maps from HeLa control (A) and AP-5 knockout cells (B, AP5Z1_KO1; C, AP5Z1_KO2), visualised by PCA. Each scatter point corresponds to a protein; proximity of proteins suggests similar fractionation behaviour and hence similar organellar association. Established marker proteins of various compartments are shown in colour. Proteins that undergo a significant shift upon AP-5 ablation are indicated with white centres. Each map combines the profiling data from 3 independent replicates (i.e., 15 data points per protein). Plots for all maps were generated in a single PCA to ensure maximum comparability (as in [15]). Projections along the first (x-axis) and third (y-axis) principal components provide the optimal visual separation of clusters; together, they account for >75% of the variability in the data. (D, E) Close-up on the endosomal cluster, where most significant protein movements occur. The control map is shown, and the shifts of retromer subunits (VPS29, VPS35, SNX2, SNX3, SNX5, SNX27), HOPS subunits (VPS16, VPS18, VPS33a, VPS39), and cation-independent mannose 6-phosphate receptor (IGF2R) in the 2 AP-5 knockout maps are indicated with arrows. These proteins undergo strikingly similar movements within the endosomal cluster, moving towards the lysosomal cluster (salmon-coloured dots). Nonmarker proteins (small grey dots) shown in the parent control map have been removed from the close-ups to enhance clarity. AP, adaptor protein; KO, knockout; PCA, principal component analysis. (PDF) [file pbio.2004411.s001.pdf]

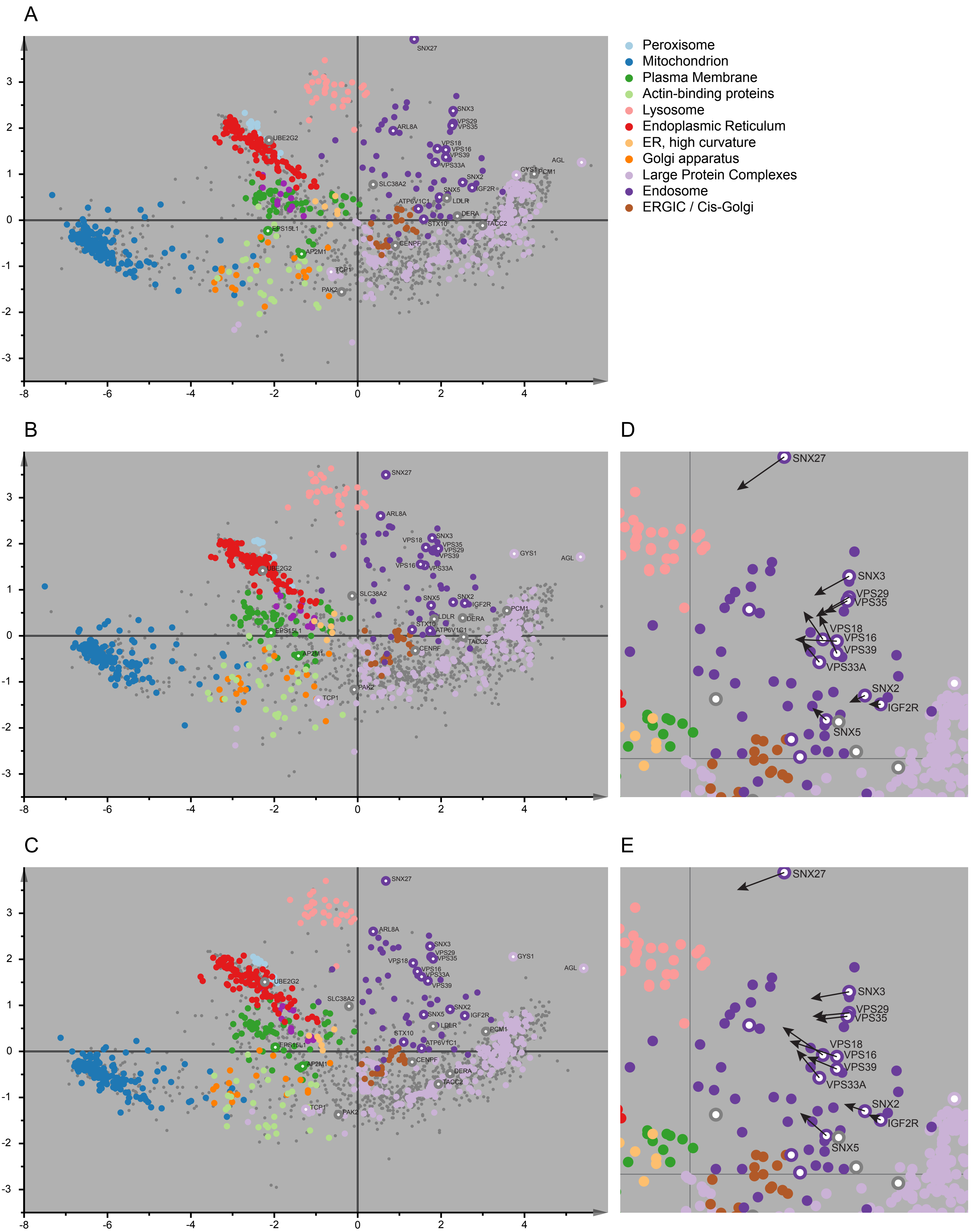
A
B
C
D
E
Peroxisome
Mitochondrion
Plasma Membrane
Actin-binding proteins
Lysosome
Endoplasmic Reticulum
ER, high curvature
Golgi apparatus
Large Protein Complexes
Endosome
ERGIC / Cis-Golgi
SNX27
SNX3
VPS29
VPS35
ARL8A
VPS18
VPS16
VPS39
VPS33A
UBE2G2
SLC38A2
SNX2
SNX5
IGF2R
LDLR
ATP6V1C1
STX10
DERA
TACC2
GYS1
PCM1
AGL
EPS15L1
AP2M1
CENPF
TCP1
PAK2
-8
-6
-4
-2
0
2
4
3
1
-1
-3
