# Supplementary figures and images for: Role of the AP-5 adaptor protein complex in late endosome-to-Golgi retrieval
Source: PLoS Biol. 2018 Jan 30;16(1):e2004411. doi: 10.1371/journal.pbio.2004411 (PMC5806898; doi:10.1371/journal.pbio.2004411)

A

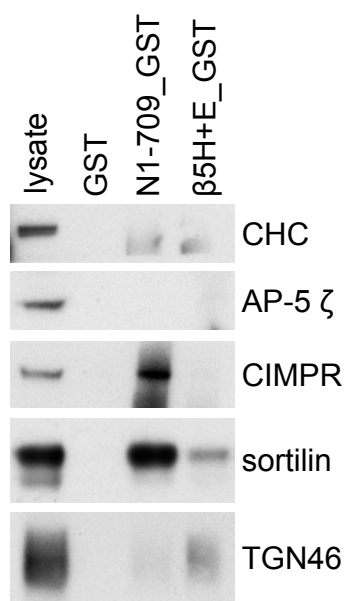

B

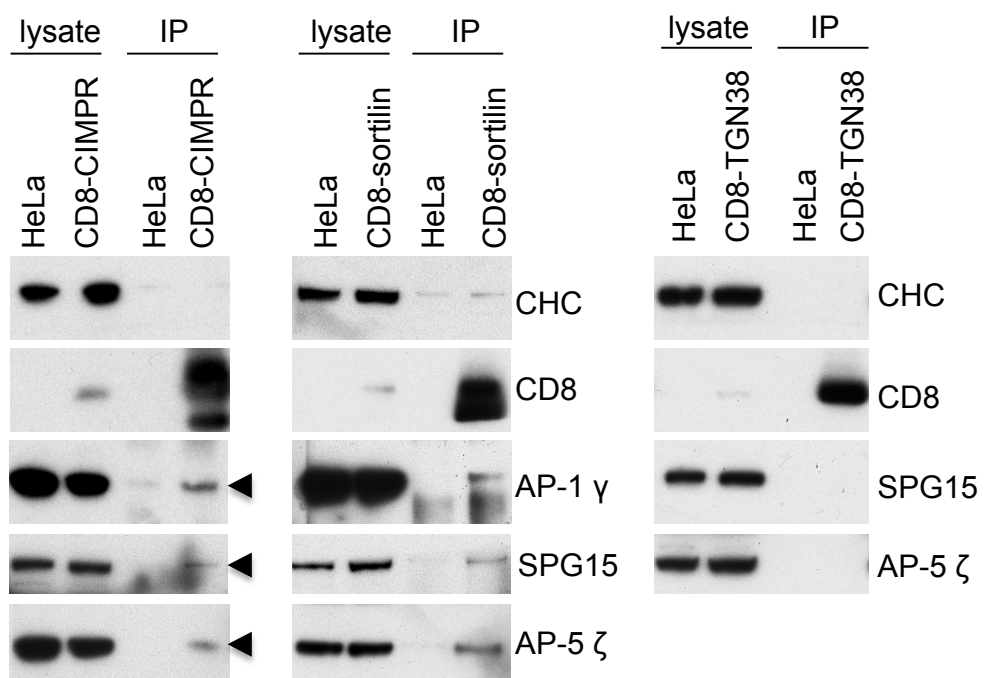

Supplement: S2 Fig — (A) Immunoprecipitations using anti-GFP were carried out on either control HeLa cells or HeLa cells stably expressing SPG15-GFP, and the blots were probed using antibodies, as shown. Both AP-5 ζ and CIMPR are specifically brought down with SPG15-GFP. Using IMAGEJ to quantify bands, we estimate that 65.9% AP-5 ζ and 0.1% CIMPR of input was pulled down by SPG15-GFP, based on 3 repeats. Blots were also probed with an antibody against CHC as a control. (B) Immunoprecipitations using anti-CD8 were carried out on either control HeLa cells or HeLa cells expressing CD8-CIMPR or CD8-sortilin, and the blots were probed using antibodies, as shown. AP-1 γ, SPG15, and AP-5 ζ are specifically brought down with CD8-CIMPR and CD8-sortilin. Using IMAGEJ to quantify bands, we estimate that 0.1% AP-1 γ, 0.15% SPG15, and 0.08% AP-5 ζ of input was pulled down by CD8-CIMPR, and 0.05% AP-1 γ, 0.15% SPG15, and 0.23% AP-5 ζ of input was pulled down by CD8-sortilin, based on 3 repeats. Blots were also probed with an antibody against CHC as a control. AP, adaptor protein; CHC, clathrin heavy chain; CIMPR, cation-independent mannose 6-phosphate receptor; SPG, spastic paraplegia gene. (PDF) [file pbio.2004411.s002.pdf]
